# Operating characteristics of unequal allocation ratios in platform trials with the staggered addition of drugs using binary endpoints

**DOI:** 10.1016/j.conctc.2025.101450

**Published:** 2025-02-17

**Authors:** Yosuke Shimizu, Ryoichi Hanazawa, Hiroyuki Sato, Akihiro Hirakawa

**Affiliations:** a Department of Clinical Biostatistics, Graduate School of Medical and Dental Sciences, Institute of Science Tokyo, Tokyo, Japan; b Biostatistics Section, Department of Data Sciences, Center for Clinical Sciences, National Center for Global Health and Medicine, Tokyo, Japan

**Keywords:** Platform trial, Allocation ratio, Drug development, Infectious diseases, Simulation

## Abstract

**Background:**

One recommendation for the allocation ratio between multiple drugs and a shared placebo control group in platform trials (PTs) is to use a $\sqrt{k}$:1 allocation ratio for the placebo group relative to the drug group, where k is the number of drug groups with ongoing patient enrollment during the trials. However, the practical utility of such unequal allocation ratios in PTs lacks adequate study.

**Methods:**

We compared the performances of equal and unequal allocation ratios through simulations to imitate practical PTs using only concurrent controls and binary endpoints for hospitalized patients with infectious diseases. The operating characteristics, including the type I error rate, power of hypothesis testing, and total sample size, were evaluated.

**Results:**

In PTs, using an unequal allocation ratio (i) results in a considerable augmentation of the total sample size and prolongs the study duration when monthly patient enrollment is low, but (ii) the target power of hypothesis testing is often preserved compared to an equal allocation ratio, even when we incorrectly specify the drug and placebo group mortality rates assumed in the sample size calculation. The average power increase using an unequal allocation ratio relative to the equal allocation ratio per 100-patient increase in the placebo group was approximately 1.9 % in the selected scenarios of our simulation studies.

**Conclusion:**

The results of the current study highlight the quantitative advantages and disadvantages of using unequal allocation ratios in PTs using only concurrent controls under the specific conditions assumed in our simulations and analyses.

## Highlights

• Unequal allocation benefits vary by enrollment speed, sample size, number of drugs, drug effect, and addition intervals.
• Low enrollment in platform trials with unequal allocation increases sample size and study duration.
• Unequal allocation preserved power better than equal allocation in platform trials, despite incorrect mortality assumptions.

## 1 Introduction

The coronavirus disease 2019 (COVID-19) pandemic has sparked a surge in randomized controlled trials (RCTs) worldwide to develop effective treatments for the disease [1]. Typically, a conventional RCT operates by pitting an investigational drug group against a control group, whether a placebo or standard of care, and enrolling a sufficient number of patients to yield statistically significant results for the primary efficacy endpoint. Historically, endpoints such as mortality in hospitalized patients, time to hospitalization, and death in non-hospitalized patients have been favored in RCTs examining COVID-19 [2,3]. However, this traditional approach, although straightforward, leads to the proliferation of individual RCTs for each investigational drug, resulting in prolonged drug development timelines, inflated patient enrollment, and a narrow focus on scientific inquiries [4,5].

In response to these limitations, the interest in platform trials (PTs) is growing, particularly in the context of infectious diseases. PTs represent a paradigm shift in evaluating multiple drugs concurrently against a common control group, such as a placebo or standard of care, within a single trial framework. These trials continually reassess various drugs for a particular disease, dynamically incorporating additions or exclusions of drugs based on interim analyses that evaluate efficacy or futility [[6], [7], [8], [9]]. Moreover, PTs introduce flexibility by allowing adaptations in study design elements, such as interim analysis, sample size re-estimation, and response-adaptive randomization rules. By amalgamating multiple investigational drugs into one trial, PTs streamline the drug development process, curtailing both the time and costs associated with launching numerous stand-alone trials.

A critical methodological consideration in designing PTs is the allocation ratio used for randomization, which directly affects the statistical power of testing, efficiency, and ethical considerations. One recommendation for the allocation ratio between multiple drugs and a shared placebo control group in PTs is to use a $\sqrt{k}$:1 allocation ratio for the placebo group relative to the drug group, where k is the number of drug groups with ongoing patient enrollment during the trial [10,11]. For example, when comparing four investigational drugs (Drugs A, B, C, and D) with a placebo, an unequal allocation ratio of (A:B:C:D:Placebo) = (1:1:1:1:2) would be recommended in PTs where all drug groups start and end simultaneously. Recently, the U.S. Food and Drug Administration (FDA) recommended that sponsors consider using a randomization ratio that allocates more patients to the control group than to each drug group, as this can increase the power for each drug versus control comparison for a given total sample size based on the findings of Chandereng et al. (2020) [12,13]. However, this unequal allocation may not be feasible under certain circumstances, such as when all the investigational drugs are not available at the start of a study. Recently, Bofill Roig et al. (2023) found optimal allocation rates when using a time-period-adjusted analysis based on regression models under PTs using a continuous endpoint that some treatment groups enter the trial at a later time point with two experimental groups and a shared control group without interim analyses [14]. We also investigated the impact of equal and unequal allocation ratios in PTs using binary endpoints with or without the staggered addition of drugs on the total development period, total sample size, and statistical operating characteristics through comprehensive simulation studies [15]. However, a more in-depth understanding of the practical operating characteristics of the unequal allocation ratio relative to the equal allocation ratio in PTs when drug group(s) enter and leave the platform over time is required.

In this study, we focused on comparing the operating characteristics (i.e., the type I error rate, power of hypothesis testing, and sample size) between equal and unequal allocation ratios in two types of trials: trials with simultaneous patient enrollment for all groups (termed multi-arm trial) and trials with the addition of drugs at different timings (termed platform trial), through simulation studies imitating frequentist PTs using binary endpoints (e.g., mortality rate) for hospitalized patients with infectious diseases [[16], [17], [18]] as registration trials for new drug application. In particular, in the early stages of drug development during a pandemic, the magnitude of the investigational drug effect on the target infection is often unknown; therefore, the magnitudes of the mortality rate in the drug and placebo groups that are assumed in the sample size determination may differ from those actually observed in the trials. Therefore, we also assessed the impact of such a misspecification (i.e., a discrepancy between the assumed and observed mortality rates) on the operating characteristics of equal and unequal allocation ratios in the multi-arm trial and platform trial.

## 2 Methods

### 2.1 Trial designs

**Fig. 1:**
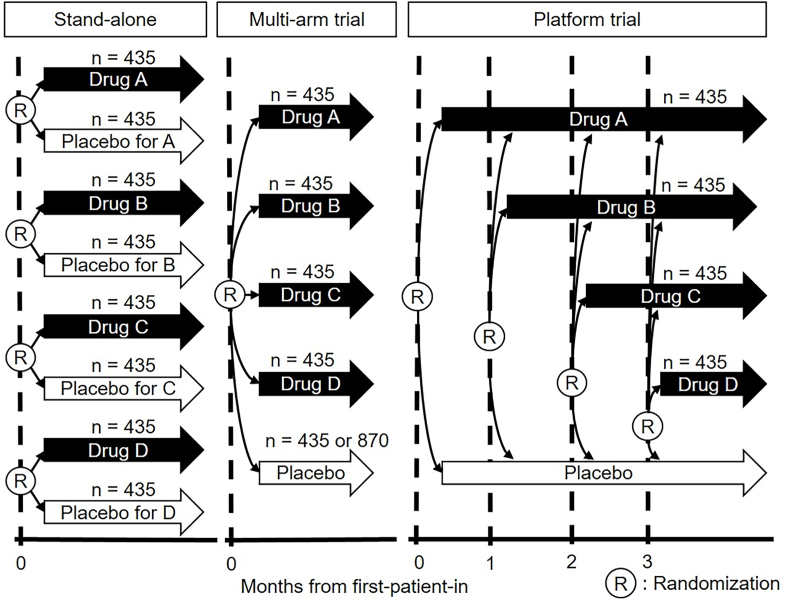
Schema of the stand-alone trial, multi-arm trial, and platform trial.

In the simulation studies, we considered to compare the mortality rates of four drugs (i.e., Drugs A, B, C, and D) with that of a placebo in an infectious disease during a pandemic and assumed the following three types of trial designs: stand-alone trial, multi-arm trial (termed MAT), and platform trial (termed PT). A stand-alone trial is defined as a RCT comparing an investigational drug group with a placebo and enrolling the required number of patients with the desired statistical power to test the statistical hypothesis of the primary efficacy endpoint of the mortality rate. We assumed that each stand-alone trial was a double-blind, placebo-controlled, phase III trial of hospitalized patients with an infectious disease and that MATs and PTs for the same population had five groups with four drug groups and a shared placebo control. In addition, we examined two situations: one in which drugs were added to PTs every month, and the other in which drugs were added to PTs at irregular intervals. Fig. 1 shows the schematic of each trial design.

We assumed two allocation ratios for MATs and PTs. One was the equal allocation ratio between the placebo and drugs. Second, for the k drug groups that enrolled patients at that time, the patients were allocated with a ratio of (A:B:C:D:Placebo) = (1:1:1:1:$\sqrt{k}$). Consequently, we compared the following seven types of trial designs: four stand-alone trials using equal allocation ratio, MAT with equal allocation ratio (termed MAT(1:1:1:1:1)), MAT with unequal allocation ratio (termed MAT(1:1:1:1:2 ($=\sqrt{4}$))), PT with equal allocation ratio where one drug is added every month (termed PT(1:1:1:1:1)), PT with unequal allocation ratio where one drug is added every month (termed PT(1:1:1:1:$\sqrt{k}$)), PT with equal allocation ratio where drugs are added at irregular intervals (termed PT-irr(1:1:1:1:1)), and PT with unequal allocation ratio where drugs are added at irregular intervals (termed PT-irr(1:1:1:1:$\sqrt{k}$)). In PT(1:1:1:1:$\sqrt{k}$) and PT-irr(1:1:1:1:$\sqrt{k}$), the allocation ratio of the placebo group ($\sqrt{k}$) varied according to the number of drug groups enrolling patients at that time. For example, when the number of drug groups that enrolled patients changed from two to three during the trials, the allocation ratio of the placebo group changed from $\sqrt{2}$ to $\sqrt{3}$. The main purpose of our simulation studies was to evaluate the increase in efficiency by increasing the allocation ratio of the placebo group to $\sqrt{k}$ compared to an equal allocation ratio, that is, a comparison of the relative efficiency of PT(1:1:1:1:$\sqrt{k}$) with PT(1:1:1:1:1) and PT-irr(1:1:1:1:$\sqrt{k}$) with PT-irr(1:1:1:1:1).

### 2.2 Sample size

We determined that 435 patients were enrolled in each drug group in the simulation studies, which was calculated based on 80 % power to detect the difference in the mortality rate between the drugs and the placebo at a two-sided 5 % alpha level using the chi-squared test (or Fisher's exact test, if necessary). For this, we assumed that the mortality rates of the effective drug and placebo groups were 5 % and 10 %, respectively, with an equal allocation ratio of 1:1 between the drug and placebo groups. Consequently, the numbers of planned total enrolled patients in the stand-alone, MAT(1:1:1:1:1), and MAT(1:1:1:1:2) were 3480 (870 patients per trial × 4 trials), 2175 (435 patients × 5 groups), and 2610 (870 patients in the placebo group + 435 patients × 4 drug groups), respectively. The total number of enrolled patients in the PT changed according to the number of patients enrolled in the entire trial per month within the range of 2175 to 3480. In our simulation studies, we fixed the number of patients in each drug group at 435. This was because the primary objective of this study was to compare the operating characteristics of MATs with those of PTs, where patient enrollment in the placebo group continued beyond 870 patients during the trials.

In the PT group, patients were non-concurrently randomized to the placebo group because patient enrollment for each drug began at different times. Including non-concurrent controls in the analysis increased the sample size and enhanced the power of hypothesis testing. However, including non-concurrent controls may introduce bias when there is a time trend, resulting in an inability to ensure comparability between groups [[19], [20], [21], [22], [23]]. In our simulation study, we considered the dynamic nature of infectious diseases like COVID-19, owing to factors such as viral mutations, initiation of vaccination, and the gradual acquisition of immunity during trial-related infection spread. To ensure a fair comparison, we specifically analyzed data from patients in the placebo group during the same period when the drug group was actively receiving treatment, termed concurrent control.

### 2.3 Simulation study

We considered five pairs of true mortality rates for the effective drug and placebo groups (Table 1) and five scenarios of true mortality rates in Drugs A, B, C, D, and placebo groups (Table 2). We also considered PTs with three (Drugs A, B, and C) and five drugs (Drugs A, B, C, D, and E) under the same mortality settings shown in Table 1, Table 2 The corresponding scenarios for true mortality rates are presented in Tables S1 and S2 of the Electronic Supplementary Material (ESM). We assumed that the true mortality rate of the drug that had no effect (termed ineffective drug) was the same as that of the placebo group. The required sample size in the previous section was calculated under the values of $\left(R_{P},R_{D}\right)=\left(10\%,5\%\right)$. Death or survival data for patients in each group were generated using a Bernoulli distribution, with the true probability of the mortality rate shown in Table 1, Table 2, S1, and S2. To imitate a more practical PT, the number of patients enrolled in the entire trial per month was simulated based on a Poisson distribution, with means of 100, 200, 300, and 400 patients. In the PT group, the enrollment of patients in the drug group proceeded until the planned number of patients was achieved. Patients in the placebo group were enrolled as long as at least one drug group remained enrolled. In PT-irr(1:1:1:1:1) and PT-irr(1:1:1:1:$\sqrt{k}$), the addition timing for Drug A was set at the start of patient enrollment, whereas the addition timings for the other drugs were generated from a uniform distribution. The ranges of the uniform distribution were from 0 to 2, 3, and 4 months when the numbers of drugs in the PTs were three, four, and five, respectively. The number of simulations was 10,000. Simulations were performed using R version 4.3.1.

**Table 1:** Five true mortality rate assumptions for simulation studies. The assumption of $\left(R_{P},R_{D}\right)=\left(10\%,5\%\right)$ was used for sample size calculation.

$R_{P}$	5 %	7.5 %	10 %	12.5 %	15 %
$R_{D}$	0 %	2.5 %	5 %	7.5 %	10 %

$R_{P}$: True mortality rate in the placebo group.

$R_{D}$: True mortality rate in the effective drug group.

**Table 2:** Five scenarios of true mortality rates in the Drug A, B, C, D, and placebo groups. The true mortality rate in the ineffective drug group was the same as that in the placebo group.

Scenarios	Placebo	Drug A	Drug B	Drug C	Drug D
1	$R_{P}$	$R_{P}$	$R_{P}$	$R_{P}$	$R_{P}$
2	$R_{P}$	$R_{P}$	$R_{P}$	$R_{P}$	$R_{D}$
3	$R_{P}$	$R_{P}$	$R_{P}$	$R_{D}$	$R_{D}$
4	$R_{P}$	$R_{P}$	$R_{D}$	$R_{D}$	$R_{D}$
5	$R_{P}$	$R_{D}$	$R_{D}$	$R_{D}$	$R_{D}$

$R_{P}$: True mortality rate in the placebo group.

$R_{D}$: True mortality rate in the effective drug group.

### 2.4 Performance indices

The following operating characteristics were calculated for the seven designs: the number of patients in the placebo group compared with each drug group, total number of patients in the placebo group, type I error rate, group-specific power for each drug, and overall power (defined as the probability of correctly declaring efficacy in one or more drugs among effective drugs). We presented the Monte Carlo standard error for the type I error rate and power in the figures.

## 3 Results

### 3.1 Number of enrolled patients in the placebo group

**Fig. 2:**
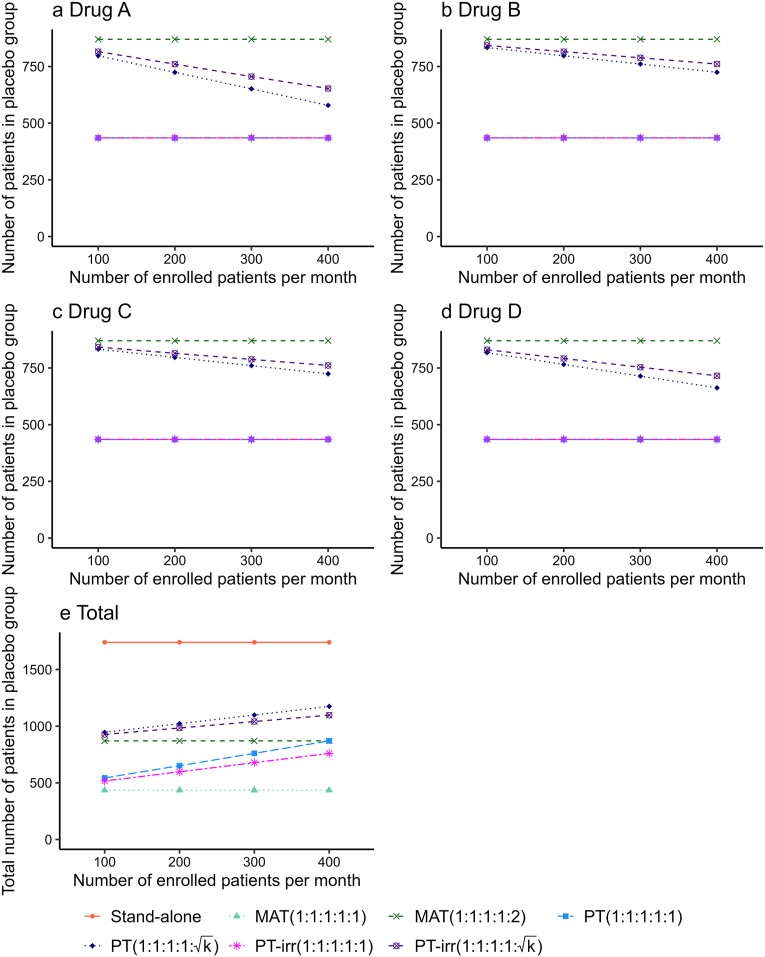
Number of patients enrolled in the placebo group compared to Drugs A, B, C, and D as well as the total number of patients in the placebo group under scenario 5 using $\left(R_{P},R_{D}\right)=\left(10\%,5\%\right)$ when the number of enrolled patients per month was between 100 and 400. The error bars are not shown because the Monte Carlo standard errors were extremely small (less than 1). Abbreviations: MAT, multi-arm trials; PT, platform trials with drugs added every month; PT-irr, platform trials with irregular intervals for drug addition.

Fig. 2 shows the number of patients enrolled in the placebo group compared to the Drugs A, B, C, and D groups (Fig. 2a–d) as well as the total number of patients enrolled in the placebo group (Fig. 2e) among the seven designs under scenario 5 using $\left(R_{P},R_{D}\right)=\left(10\%,5\%\right)$, when the number of enrolled patients per month was between 100 and 400. The number of patients in the placebo group for each drug group was identical to 435 in the stand-alone, MAT(1:1:1:1:1), PT(1:1:1:1:1), and PT-irr(1:1:1:1:1) and 870 in the MAT(1:1:1:1:2) group, as determined by the sample size calculation described in the previous section. According to Fig. 2a–d, the number of patients in the placebo group of PT(1:1:1:1:$\sqrt{k}$) and PT-irr(1:1:1:1:$\sqrt{k}$) decreased with an increasing number of enrolled patients per month. For each drug group, the number of patients in the placebo group for PT-irr(1:1:1:1:$\sqrt{k}$) was greater than that for PT(1:1:1:1:$\sqrt{k}$), and the difference increased as the number of patients enrolled per month increased. As shown in Fig. 2e, with an increasing number of enrolled patients per month, the total number of patients enrolled in the placebo group in the PT(1:1:1:1:1) and PT(1:1:1:1:$\sqrt{k}$) groups increased, but the difference between them gradually decreased. That is, when patient enrollment per month was low, employing PT(1:1:1:1:$\sqrt{k}$) led to a substantial increase in the total sample size and extended the study duration. Similar trends were observed in the total number of patients enrolled in the placebo group for PT-irr(1:1:1:1:1) and PT-irr(1:1:1:1:$\sqrt{k}$). This is the cost of optimizing the statistical efficiency of drug effect inference through unequal allocation.

### 3.2 Type I error rate and power

Because of the increase in the total sample size in PT(1:1:1:1:$\sqrt{k}$) and PT-irr(1:1:1:1:$\sqrt{k}$), we further investigated the impact of increasing the sample size on the type I error rate and the power of hypothesis testing under scenarios 1–5 of the true mortality rate in PT(1:1:1:1:$\sqrt{k}$). We first assessed whether the type I error rate and power under scenarios 1–5 with $\left(R_{P},R_{D}\right)=\left(10\%,5\%\right)$ used in the sample size calculation were maintained at the nominal values of 5 % and 80 %, respectively, among the seven designs we compared (Fig. S1 c1–c5). According to Fig. S1 c1–c5, all designs maintained type I error rates at approximately 5 % and group-specific powers at over 80 %. In the other values of $\left(R_{P},R_{D}\right)$, type I error rates were also maintained at approximately 5 % for all designs (Fig. S1 a1–b5 and d1–e5).

When the true mortality rate in the placebo group, $R_{P}$, was ≤10 %, the group-specific power (or overall power) was exceeded the target level of 80 % in all cases among the seven designs because the variance of mortality rates became small (e.g., range of 93.1 %–100 % for stand-alone trial; 93.1 %–100 % for MAT(1:1:1:1:1); 97.6 %–100 % for MAT(1:1:1:1:2); 93.4 %–100 % for PT(1:1:1:1:1); 96.0 %–100 % for PT(1:1:1:1:$\sqrt{k}$); 93.2 %–100 % for PT-irr(1:1:1:1:1); 96.4 %–100 % for PT-irr(1:1:1:1:$\sqrt{k}$)). PT(1:1:1:1:$\sqrt{k}$) exhibited a higher group-specific power to PT(1:1:1:1:1) because of increase the number of patients in placebo group when decreasing the number of enrolled patients per month, but showed a similar power with an increasing number of enrolled patients.

By contrast, when the true mortality rate in the placebo group, $R_{P}$, was 12.5 %, the group-specific power fell below the target level of 80 % in many cases in the stand-alone trial, MAT(1:1:1:1:1), PT(1:1:1:1:1), and PT-irr(1:1:1:1:1) (Fig. S1 d2–d5), because the variance in mortality rates increased. However, MAT(1:1:1:1:2), PT(1:1:1:1:$\sqrt{k}$) and PT-irr(1:1:1:1:$\sqrt{k}$) under the specific scenarios often maintained the nominal group-specific power of approximately 80 % (68.9 %–70.2 % in stand-alone, 69.5 %–70.2 % in MAT(1:1:1:1:1), 80.0 %–80.2 % in MAT(1:1:1:1:2), 69.1 %–70.1 % in PT(1:1:1:1:1), 75.1 %–80.1 % in PT(1:1:1:1:$\sqrt{k}$), 69.1 %–70.3 % in PT-irr(1:1:1:1:1), and 76.5 %–80.5 % in PT-irr(1:1:1:1:$\sqrt{k}$)). When the true mortality rate in the placebo group, $R_{P}$, was 15 %, the group-specific power fell below 80 % among the seven designs, but PT(1:1:1:1:$\sqrt{k}$) and PT-irr(1:1:1:1:$\sqrt{k}$) showed higher power than PT(1:1:1:1:1) and PT-irr(1:1:1:1:1) respectively (60.3 %–61.4 % in stand-alone, 61.0 %–61.7 % in MAT(1:1:1:1:1), 71.9 %–72.5 % in MAT(1:1:1:1:2), 60.2 %–61.6 % in PT(1:1:1:1:1), 66.5 %–71.6 % in PT(1:1:1:1:$\sqrt{k}$), 60.1 %–61.3 % in PT-irr(1:1:1:1:1), and 68.2 %–72.0 % in PT-irr(1:1:1:1:$\sqrt{k}$)). Thus, PT(1:1:1:1:$\sqrt{k}$) was often superior to PT(1:1:1:1:1) (e.g., 65 % among all simulated trials we conducted) in terms of maintaining the nominal group-specific power when we incorrectly specified the (higher) mortality rates in the drug and placebo groups assumed in the sample size calculation.

For all the true mortality rates we assumed in the placebo group, PT-irr(1:1:1:1:$\sqrt{k}$) presented a slightly higher group-specific power compared to PT(1:1:1:1:$\sqrt{k}$) because the addition of the last drug in PT-irr(1:1:1:1:$\sqrt{k}$) was earlier than that in PT(1:1:1:1:$\sqrt{k}$) and subsequently the relatively increased overlap of the patient enrollment periods between the drugs increased the number of patients in the concurrent control group in each drug group.

### 3.3 Difference in type I error rate/power between equal and unequal allocation ratios

**Fig. 3:**
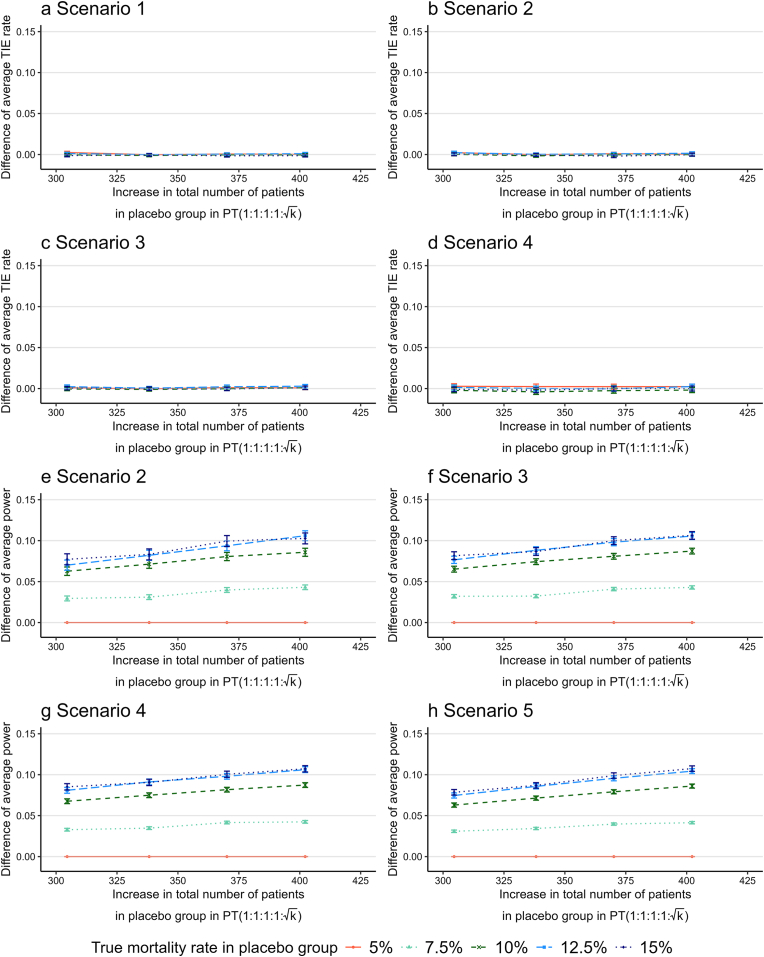
Difference in the average type I error rate (or power) across four drug groups between PT(1:1:1:1:$\sqrt{k}$) and PT(1:1:1:1:1) (i.e., PT(1:1:1:1:$\sqrt{k}$) − PT(1:1:1:1:1)) for the corresponding increase in total number of patients in the placebo group of PT(1:1:1:1:$\sqrt{k}$), assuming that the true mortality rate for the placebo group was 5 %, 7.5 %, 10 %, 12.5 %, and 15 %. The Monte Carlo standard errors for the difference in the average type I error rate (or power) are presented as error bars. Abbreviations: PT, platform trials with drugs added every month; TIE, type I error.

Fig. 3 shows the difference in the average type I error rate (or group-specific power) across four drug groups between PT(1:1:1:1:$\sqrt{k}$) and PT(1:1:1:1:1) (i.e., PT(1:1:1:1:$\sqrt{k}$) − PT(1:1:1:1:1)) for the corresponding increase in the total number of patients in the placebo group observed in PT(1:1:1:1:$\sqrt{k}$) shown in Fig. 2 under scenario 5 using $\left(R_{P},R_{D}\right)=\left(10\%,5\%\right)$ when assuming that the true mortality rate for the placebo group, $R_{P}$, was 5 %, 7.5 %, 10 %, 12.5 %, and 15 %. The results for Drug A, B, C, and D groups are shown in Fig. S2 of ESM.

The average type I error rate of PT(1:1:1:1:$\sqrt{k}$) was nearly the same as that of PT(1:1:1:1:1) for all the true mortality rates, irrespective of the increase in sample size of the placebo group (Fig. 3a–d). The average power increase of PT(1:1:1:1:$\sqrt{k}$) relative to PT(1:1:1:1:1) by increasing sample size in the placebo group was 1.9 % (95 % confidence interval [1.5 %, 2.3 %]) per 100-patient increase across the scenarios 2–5 (Fig. 3e–h) based on the results of linear regression analysis. Notably, no increase in power was observed when the assumed mortality rate in the placebo group was 5 % because both PT(1:1:1:1:$\sqrt{k}$) and PT(1:1:1:1:1) maintained a group-specific power of 100 % in the selected scenarios in our simulation studies.

We also examined the differences in the average type I error rate (or group-specific power) between PT-irr(1:1:1:1:$\sqrt{k}$) and PT-irr(1:1:1:1:1) (i.e., PT-irr(1:1:1:1:$\sqrt{k}$) − PT-irr(1:1:1:1:1)) for the corresponding increase in the total number of patients in the placebo group (Fig. S3). The average type I error rates between the two designs were almost the same, and the average power increase per 100-patient increase in the placebo group was mostly 2.0 % (95 % confidence interval [1.6 %, 2.4 %]). Therefore, adding drugs at irregular intervals had little effect on the average type I error rate and group-specific power changes resulting from an unequal allocation ratio.

### 3.4 Effect of number of drug groups on type I error rate and power in platform trials

**Fig. 4:**
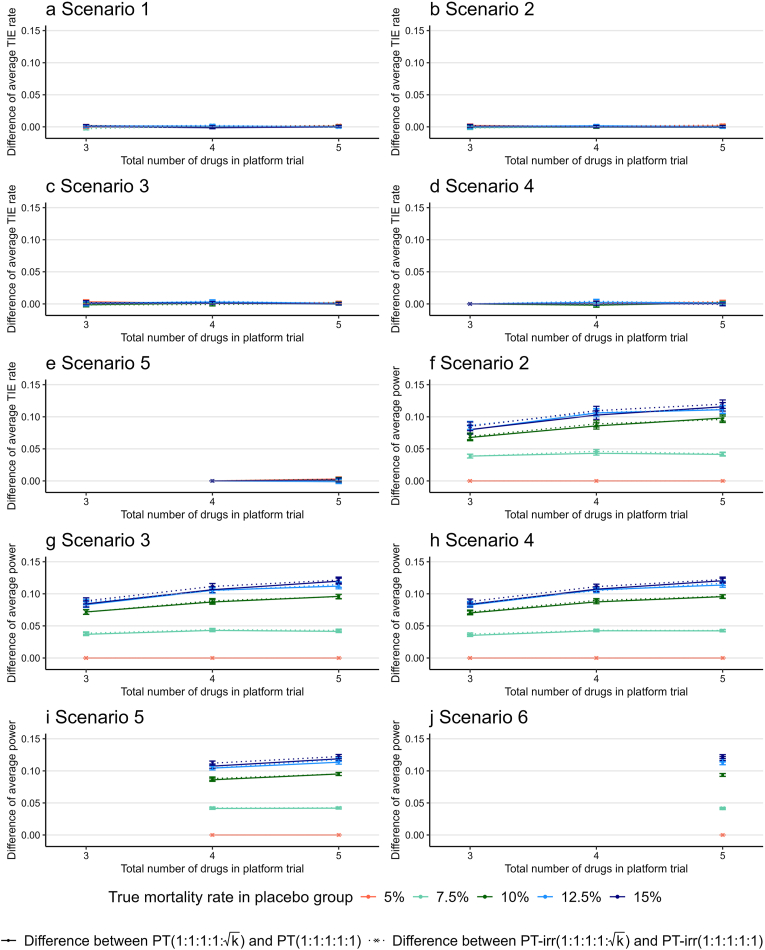
Difference in the average type I error rate and average group-specific power across the drug groups included in the PT between PT(1:1:1:1:$\sqrt{k}$) and PT(1:1:1:1:1) (e.g., PT(1:1:1:1:$\sqrt{k}$) − PT(1:1:1:1:1)) and between PT-irr(1:1:1:1:$\sqrt{k}$) and PT-irr(1:1:1:1:1) (e.g., PT-irr(1:1:1:1:$\sqrt{k}$) − PT-irr(1:1:1:1:1)) when the total number of drugs was three, four, or five with an average enrollment of 100 patients per month and the true mortality rate for the placebo group, $R_{P}$, ranging from 5 % to 15 %. The number of scenarios depends on the number of drugs included in the PT (see Tables 2, S1, and S2). Therefore, the results for PTs with three or four drugs are not displayed in scenarios 5 and 6. The Monte Carlo standard errors for the difference in the average type I error rate (or group-specific power) are presented as error bars. Abbreviations: PT, platform trials with drugs added every month; PT-irr, platform trials with irregular intervals for drug addition; TIE, type I error.

Fig. 4 shows the difference in the average type I error rate (Fig. 4a–e) and average group-specific power (Fig. 4f–j) across the drug groups included in the trial between PT(1:1:1:1:$\sqrt{k}$) and PT(1:1:1:1:1) (e.g., PT(1:1:1:1:$\sqrt{k}$) − PT(1:1:1:1:1)) and between PT-irr(1:1:1:1:$\sqrt{k}$) and PT-irr(1:1:1:1:1) (e.g., PT-irr(1:1:1:1:$\sqrt{k}$) − PT-irr(1:1:1:1:1)) when the total number of drugs was three, four, or five with an average enrollment of 100 patients per month and the true mortality rate for the placebo group, $R_{P}$, ranging from 5 % to 15 %. These differences in the average type I error rates were almost zero for all scenarios, whereas the differences in the average group-specific power tended to increase gradually as the number of drugs increased, except when the true mortality rate for the placebo group was 5 %. This is because, as described in the previous section, all four designs maintained 100 % group-specific power when the true mortality rate for the placebo group was 5 %.

Figs. S4–S6 present the results for when the average enrollment rates were 200, 300, and 400 patients per month, confirming that similar tendencies were observed regardless of the enrollment rate. We also evaluated the increase in the average power per 100-patient increase in the placebo group using linear regression analysis when the number of drug arms was three or five. For three drug arms, PT(1:1:1:$\sqrt{k}$) increased by 1.1 % (95 % confidence interval [0.8 %, 1.4 %]) and PT-irr(1:1:1:$\sqrt{k}$) increased by 1.3 % (95 % confidence interval [1.0 %, 1.6 %]). For five drug arms, PT(1:1:1:1:1:$\sqrt{k}$) increased by 2.1 % (95 % confidence interval [1.7 %, 2.6 %]) and PT-irr(1:1:1:1:1:$\sqrt{k}$) increased by 2.3 % (95 % confidence interval [1.8 %, 2.7 %]). The average power per 100 additional patients in the placebo group obtained using the unequal allocation ratio increased as the number of drugs tested in the PT increased.

## 4 Discussion

The current study compared the operating characteristics, including the type I error rate, power of hypothesis testing, and total sample size, between equal and unequal allocation ratios in the context of a MAT and a PT with the staggered addition of a drug for an infectious disease along with stand-alone trials through practical simulation studies. Moreover, it investigated the benefit of using the unequal allocation ratio for the control group of $\sqrt{k}$ relative to the equal allocation ratio between multiple drugs and one shared placebo group under several scenarios regarding the number of drugs and the intervals between drug additions.

Our simulation studies revealed several findings regarding unequal allocation ratios. When patient enrollment per month is low, implementing PT using an unequal allocation ratio results in considerable augmentation of the total sample size and prolongs the duration of the study. We believe that this cost (i.e., an increase in sample size and study period) represents a necessary compromise for optimizing the statistical efficiency in inferring drug effects using an unequal allocation ratio. However, PT using an unequal allocation ratio often outperformed PT using an equal allocation ratio in preserving the nominal (group-specific) power when we incorrectly specified the mortality rates in the drug and placebo groups assumed in the sample size calculation. This feature would be useful when there is insufficient information on the efficacy of the investigational drug in the early stages of drug development during a pandemic. Furthermore, the average power increase of PT using an unequal allocation ratio relative to PT using an equal allocation ratio per 100-patient increase in the placebo group was approximately 1.9 %. These trade-off results highlight the quantitative advantages and disadvantages of using unequal allocation ratios in PT.

We also investigated a PT with irregular intervals for drug addition, where new arms were introduced at random intervals. The results of simulation studies demonstrated that these irregular intervals for drug addition do not significantly affect the extent to which an unequal allocation ratio enhances the average power. Furthermore, in PTs with irregular intervals for drug addition, the last drug was added earlier than in fixed-interval PTs (i.e., PT(1:1:1:1:1) and PT(1:1:1:1:$\sqrt{k}$)) under our simulation settings. This increased the overlap in the patient enrollment periods between the drug groups, resulting in more concurrent controls and a slight improvement in the average power.

We also evaluated the operating characteristics of PTs when the total number of drugs tested in the PT was three or five. As the number of drug arms increases, the proportion of patients assigned to the shared placebo group increases under unequal allocation, resulting in improvements in the average power compared to equal allocation. These findings suggest that unequal allocation becomes increasingly beneficial as additional drug arms are introduced, provided patient enrollment is sufficient.

In addition to the consideration of optimal allocation ratios in PTs, the utility of the response adaptive randomization (RAR) technique in PTs can be discussed [[24], [25], [26], [27]]. The RAR updates the randomization probability using the accrual trial data, which is often used to minimize the number of patients randomized to inferior treatments (or placebo) and increase the amount of information about better treatments. However, fewer randomizations to the inferior treatments may result in lower accuracy of statistical estimation in between-group comparisons compared to 1:1 allocation, and further temporal trends during the study may result in bias and inflation of the type I error rate [25,28]. A comparative study between unequal allocation ratio and RAR in PTs is warranted.

While our simulation study provides valuable insights into the design and operation of PTs during a pandemic, several limitations must be acknowledged. First, our simulation studies did not include interim analyses. Interim analyses are crucial for making early decisions about the efficacy or futility of drugs, thereby reducing risks to participants and improving trial efficiency. We recognize this limitation and plan to incorporate interim analyses into future simulations to better align with standard practices in MATs and PTs. Second, our study employed a frequentist approach, primarily chosen for its simplicity and widespread recognition. However, many modern PTs utilize Bayesian methods, which offer significant advantages in incorporating prior information and providing probabilistic interpretations of results. Although we selected a frequentist design for its regulatory acceptability, future iterations of our study will explore Bayesian designs to align more closely with current methodological trends and enhance decision-making processes. Third, the use of a binary endpoint in our simulations, particularly when the endpoint is death, represents another limitation. In the context of a pandemic, where various factors can influence trial outcomes, a survival endpoint could provide more detailed and informative results. Future simulations will consider survival endpoints to address this limitation and improve the relevance and accuracy of our findings. In addition, if the investigational drug demonstrates a lower mortality rate or the placebo group exhibits a higher mortality rate than assumed in our simulation studies, the variance across drug arms may become more heterogeneous. This increased heterogeneity could undermine the robustness of our findings and weaken their generalizability to broader clinical settings. Finally, our choice of including only concurrent controls in the analysis could lead to a larger total sample size and a prolonged study duration, particularly when patient enrollment is slow, although the use of only concurrent controls generally minimizes the bias compared to analyses incorporating both concurrent and nonconcurrent controls, which may be influenced by time trend effects. This trade-off is particularly significant in pandemic settings, where the urgency of developing new treatments must be balanced with the need to rigorously control bias. Researchers should therefore carefully balance these competing priorities when designing PTs.

## 5 Conclusion

We demonstrated the quantitative advantages and disadvantages of using unequal allocation ratios in PTs using only concurrent controls under the specific conditions assumed in our simulations and analyses through simulation studies with selected scenarios. Notably, the benefit of the unequal allocation ratio relative to the equal allocation ratio would vary depending on the speed of patient enrollment, planned sample size in each group, true drug effect, number of drugs tested in the PT, and interval of staggered addition of drugs, that is, the speed of the spread of infection. Understanding the significance of considering these factors is crucial when implementing an unequal allocation ratio.

## CRediT authorship contribution statement

**Yosuke Shimizu:** Writing – review & editing, Writing – original draft, Visualization, Validation, Software, Project administration, Methodology, Investigation, Formal analysis, Conceptualization. **Ryoichi Hanazawa:** Writing – review & editing, Writing – original draft, Visualization, Validation, Software, Project administration, Methodology, Investigation, Formal analysis, Conceptualization. **Hiroyuki Sato:** Writing – review & editing, Supervision. **Akihiro Hirakawa:** Writing – review & editing, Writing – original draft, Supervision, Resources, Funding acquisition, Conceptualization.

## Funding statement

This study was partially supported by the 10.13039/100009619Japan Agency for Medical Research and Development (grant number JP24mk0121268).

## Declaration of competing interest

The authors declare that they have no known competing financial interests or personal relationships that could have appeared to influence the work reported in this paper.
