# Supplementary material for: Operating characteristics of unequal allocation ratios in platform trials with the staggered addition of drugs using binary endpoints
Source: Contemp Clin Trials Commun. 2025 Feb 17;44:101450. doi: 10.1016/j.conctc.2025.101450 (PMC11889599; doi:10.1016/j.conctc.2025.101450)

**Electronic supplementary material**

**Operating characteristics of unequal allocation ratios in platform trials with the staggered addition of drugs using binary endpoints**

Yosuke Shimizu, Ryoichi Hanazawa, Hiroyuki Sato, Akihiro Hirakawa^*^.

^*^ Corresponding author

Akihiro Hirakawa, PhD

Department of Clinical Biostatistics, Graduate School of Medical and Dental Sciences, Institute of Science Tokyo, 1-5-45 Yushima, Bunkyo-ku, Tokyo 113-8510, Japan.

Email: [a-hirakawa.crc@tmd.ac.jp](mailto:a-hirakawa.crc@tmd.ac.jp)

Journal: Contemporary Clinical Trials Communications

**Table S1.** Four scenarios of true mortality rates in the Drug A, B, C, and placebo groups. The true mortality rate in the ineffective drug group was the same as that in the placebo group.

| Scenarios | Placebo | Drug A | Drug B | Drug C |
| --- | --- | --- | --- | --- |
| 1 | $R_{P}$ | $R_{P}$ | $R_{P}$ | $R_{P}$ |
| 2 | $R_{P}$ | $R_{P}$ | $R_{P}$ | $R_{D}$ |
| 3 | $R_{P}$ | $R_{P}$ | $R_{D}$ | $R_{D}$ |
| 4 | $R_{P}$ | $R_{D}$ | $R_{D}$ | $R_{D}$ |

$R_{P}$: True mortality rate in the placebo group

$R_{D}$: True mortality rate in the effective drug group

**Table S2.** Six scenarios of true mortality rates in the Drug A, B, C, D, E, and placebo groups. The true mortality rate in the ineffective drug group was the same as that in the placebo group.

| Scenarios | Placebo | Drug A | Drug B | Drug C | Drug D | Drug E |
| --- | --- | --- | --- | --- | --- | --- |
| 1 | $R_{P}$ | $R_{P}$ | $R_{P}$ | $R_{P}$ | $R_{P}$ | $R_{P}$ |
| 2 | $R_{P}$ | $R_{P}$ | $R_{P}$ | $R_{P}$ | $R_{P}$ | $R_{D}$ |
| 3 | $R_{P}$ | $R_{P}$ | $R_{P}$ | $R_{P}$ | $R_{D}$ | $R_{D}$ |
| 4 | $R_{P}$ | $R_{P}$ | $R_{P}$ | $R_{D}$ | $R_{D}$ | $R_{D}$ |
| 5 | $R_{P}$ | $R_{P}$ | $R_{D}$ | $R_{D}$ | $R_{D}$ | $R_{D}$ |
| 6 | $R_{P}$ | $R_{D}$ | $R_{D}$ | $R_{D}$ | $R_{D}$ | $R_{D}$ |

$R_{P}$: True mortality rate in the placebo group

$R_{D}$: True mortality rate in the effective drug group

**Figure S1.** The type I error rate and power for each group, and overall power, with the Monte Carlo standard errors shown as error bars in 10,000 simulated trials for four-drug trials.
Abbreviations: MAT, multi-arm trials; PT, platform trials with drugs added every month; PT-irr, platform trials with irregular intervals for drug addition.

(a1) ($R_{P}, R_{D}$) = (5%, 0%) in scenario 1


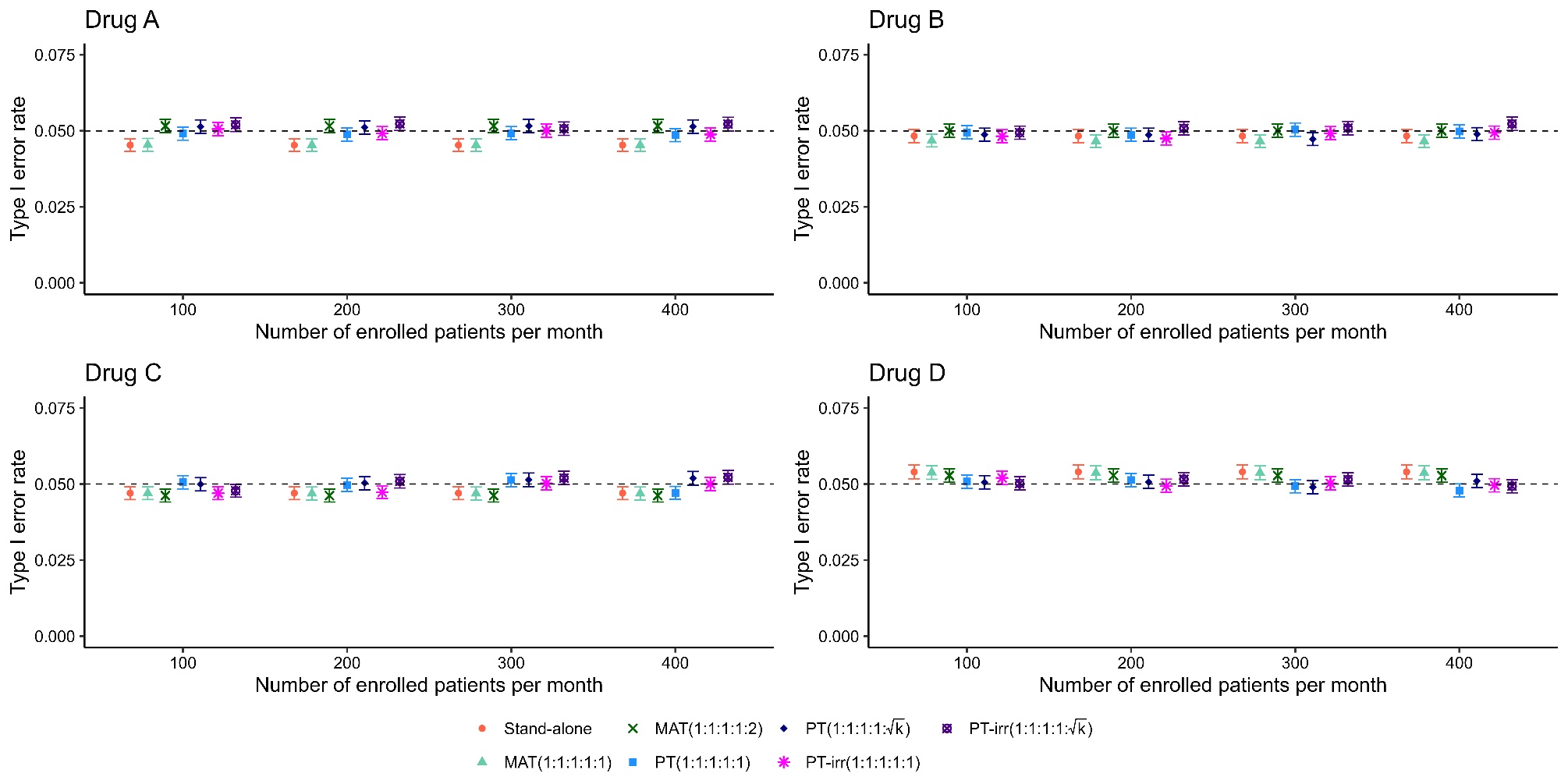


(a2) ($R_{P}, R_{D}$) = (5%, 0%) in scenario 2


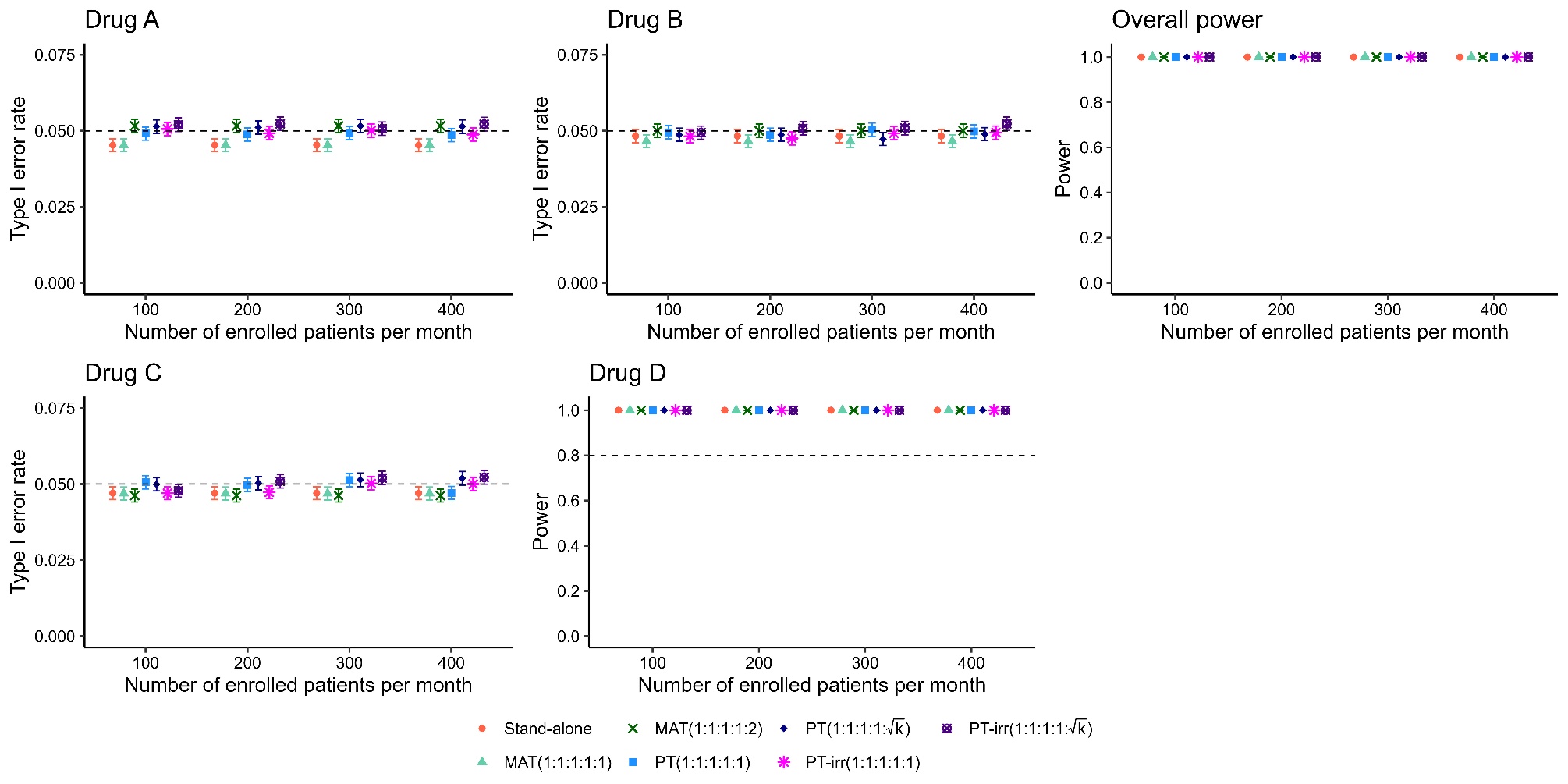


(a3) ($R_{P}, R_{D}$) = (5%, 0%) in scenario 3


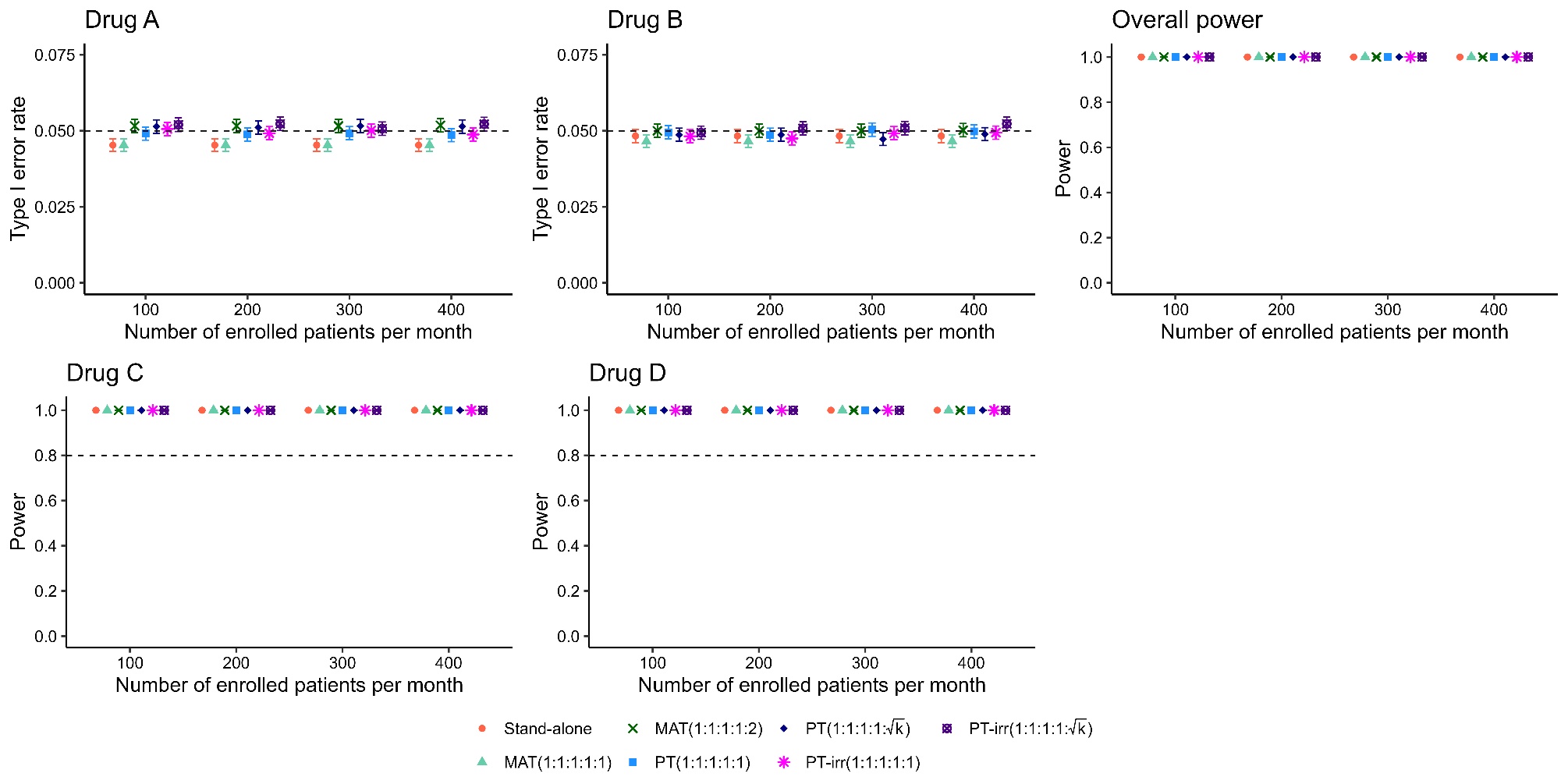


(a4) ($R_{P}, R_{D}$) = (5%, 0%) in scenario 4


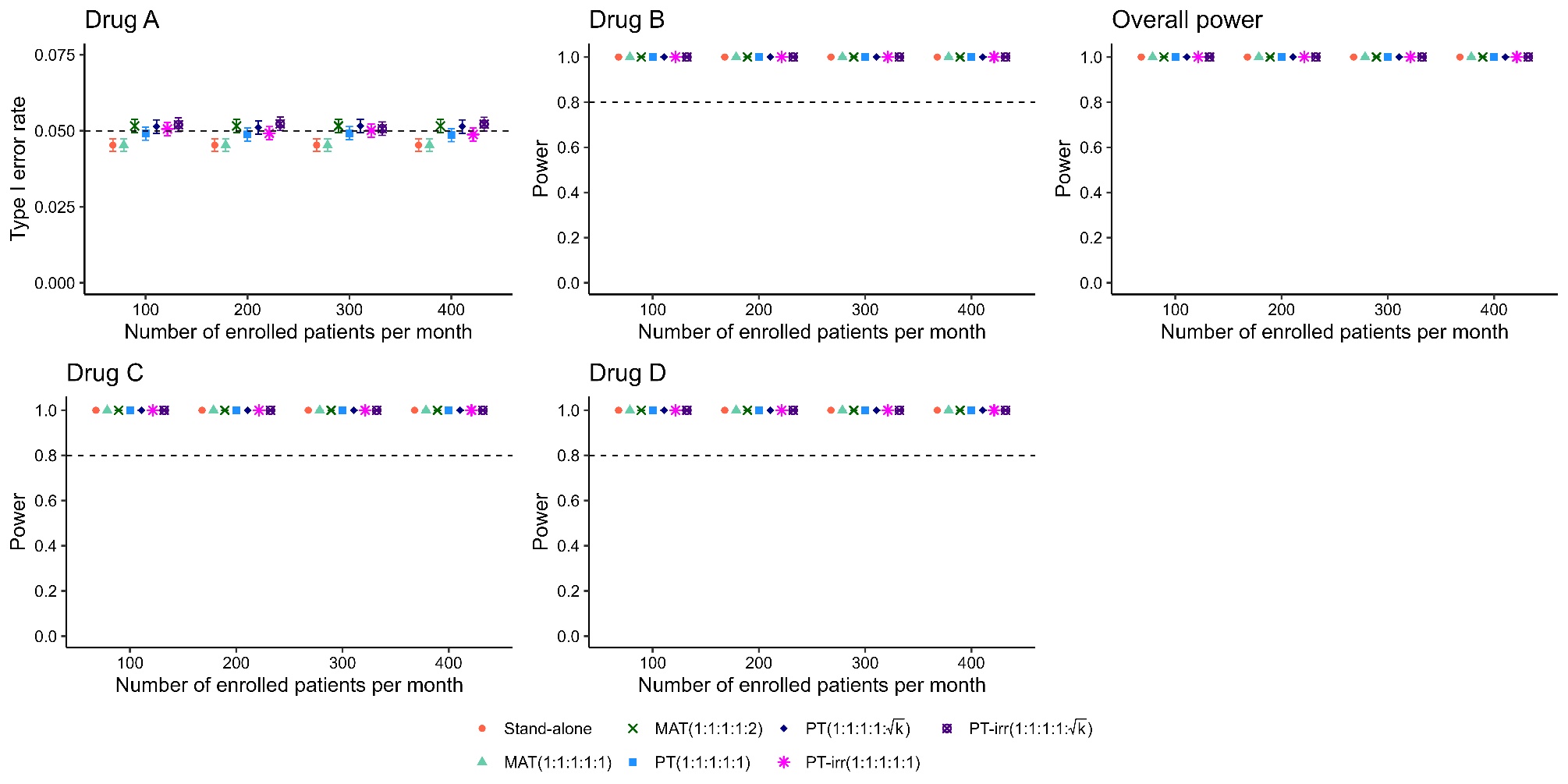


(a5) ($R_{P}, R_{D}$) = (5%, 0%) in scenario 5


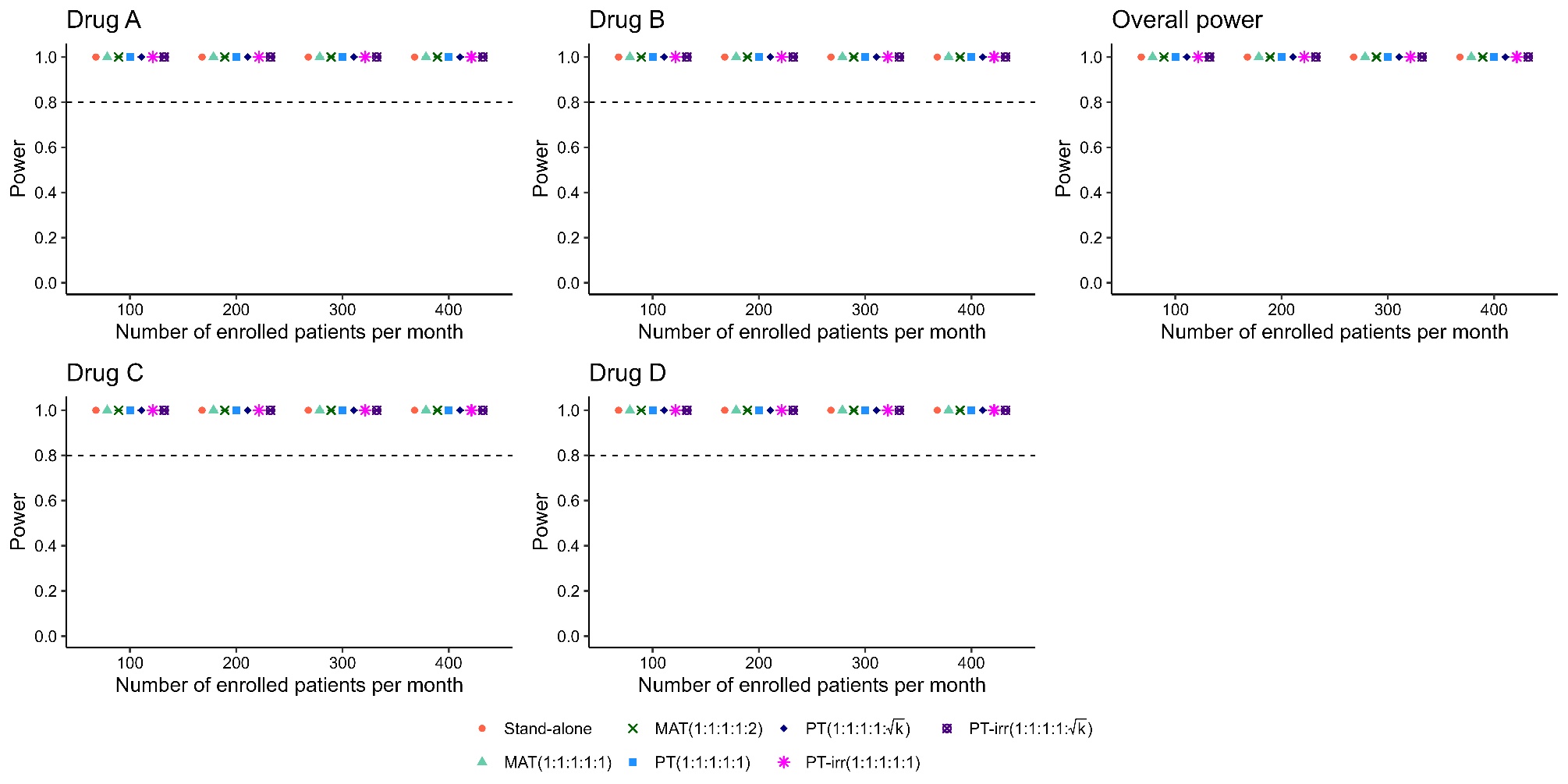


(b1) ($R_{P}, R_{D}$) = (7.5%, 2.5%) in scenario 1


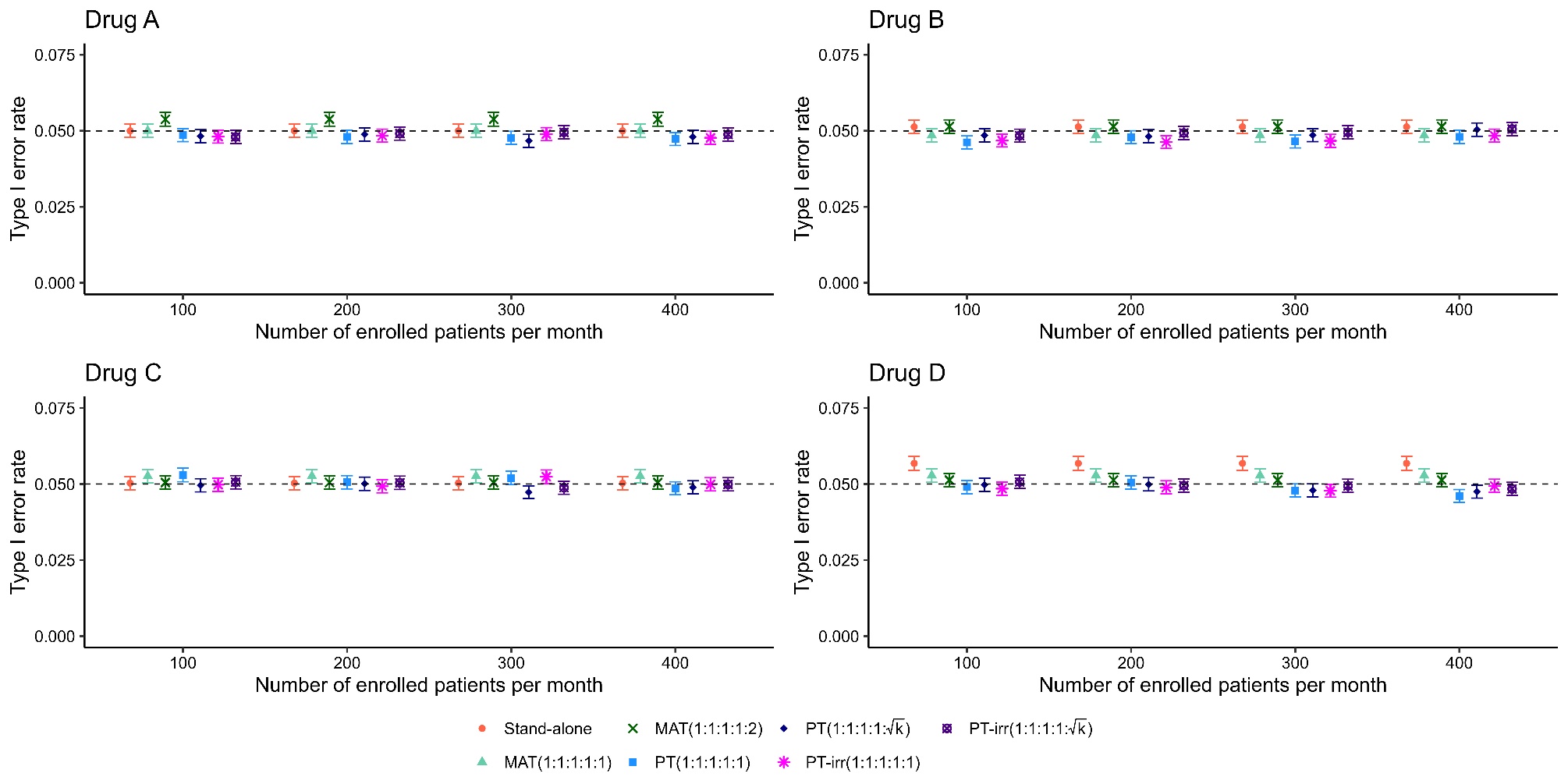


(b2) ($R_{P}, R_{D}$) = (7.5%, 2.5%) in scenario 2


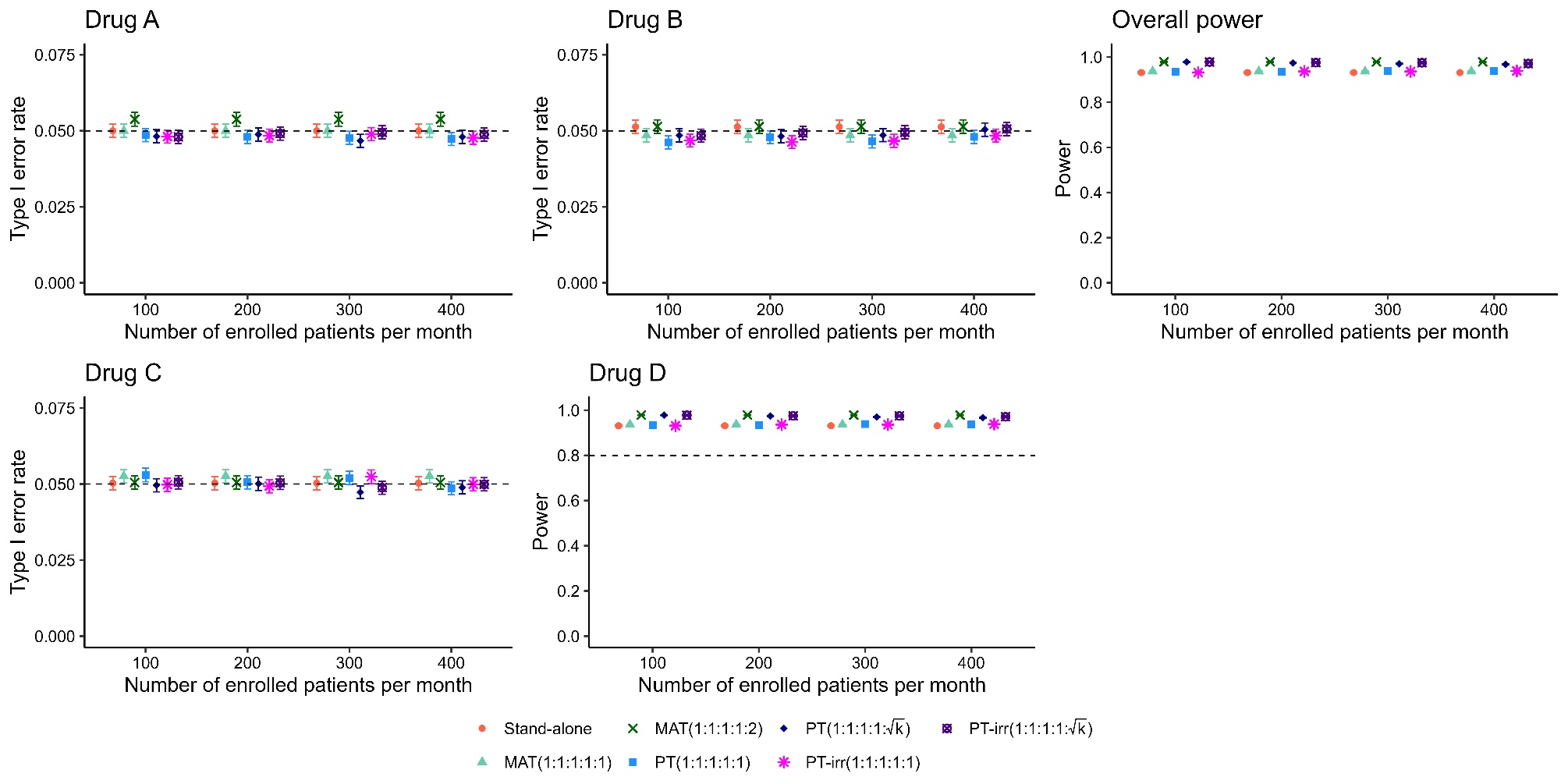


(b3) ($R_{P}, R_{D}$) = (7.5%, 2.5%) in scenario 3


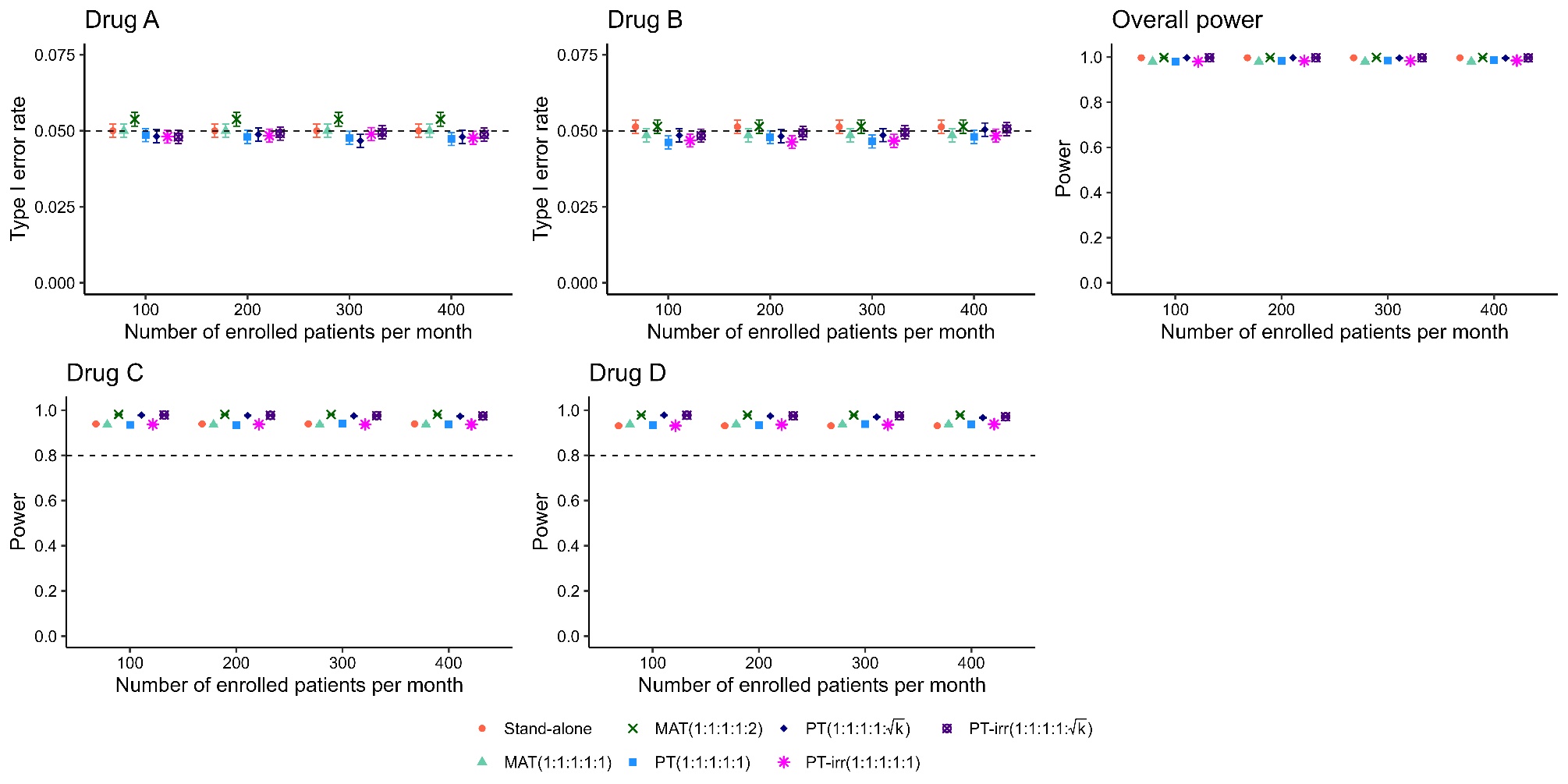


(b4) ($R_{P}, R_{D}$) = (7.5%, 2.5%) in scenario 4


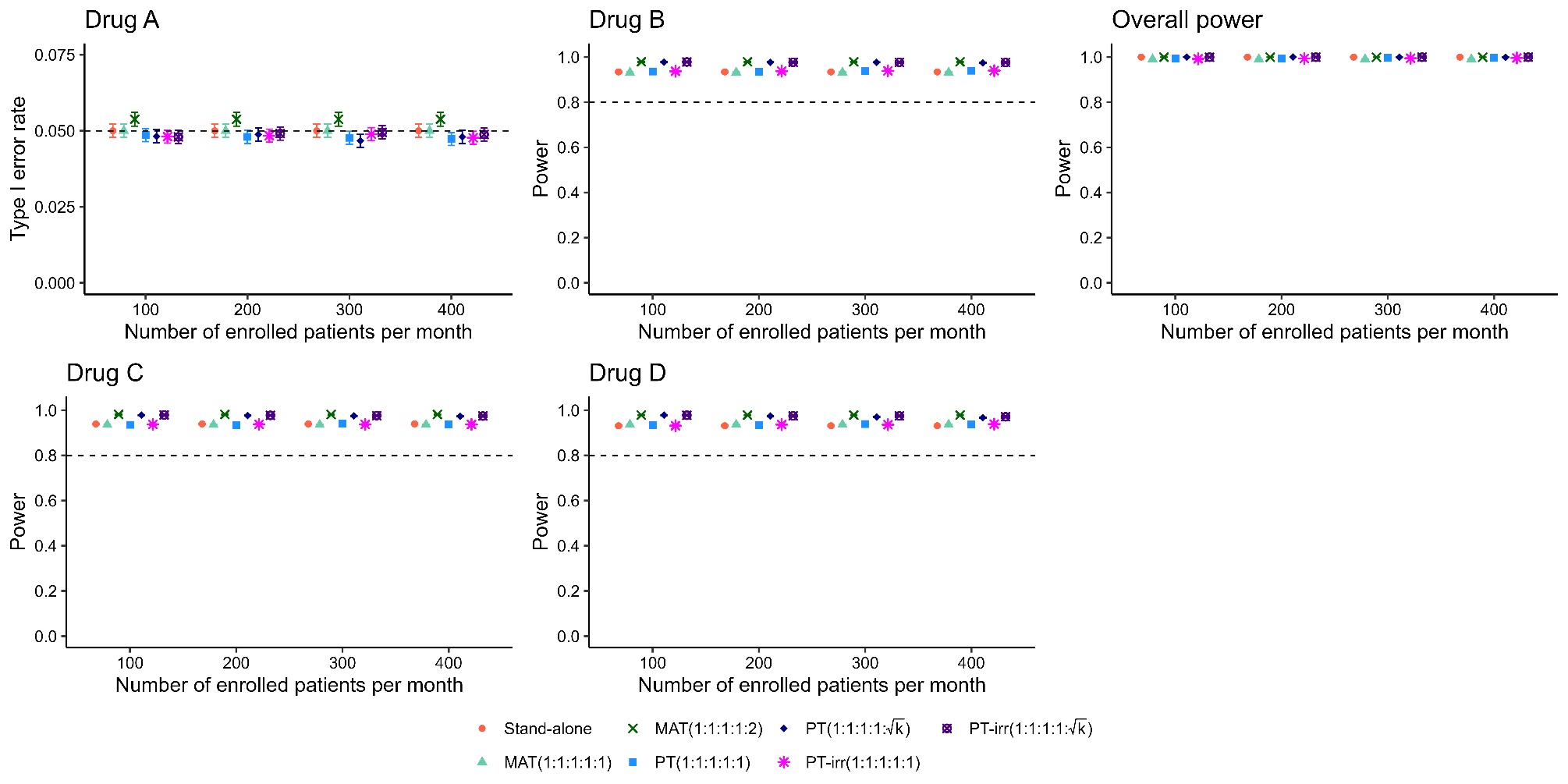


(b5) ($R_{P}, R_{D}$) = (7.5%, 2.5%) in scenario 5


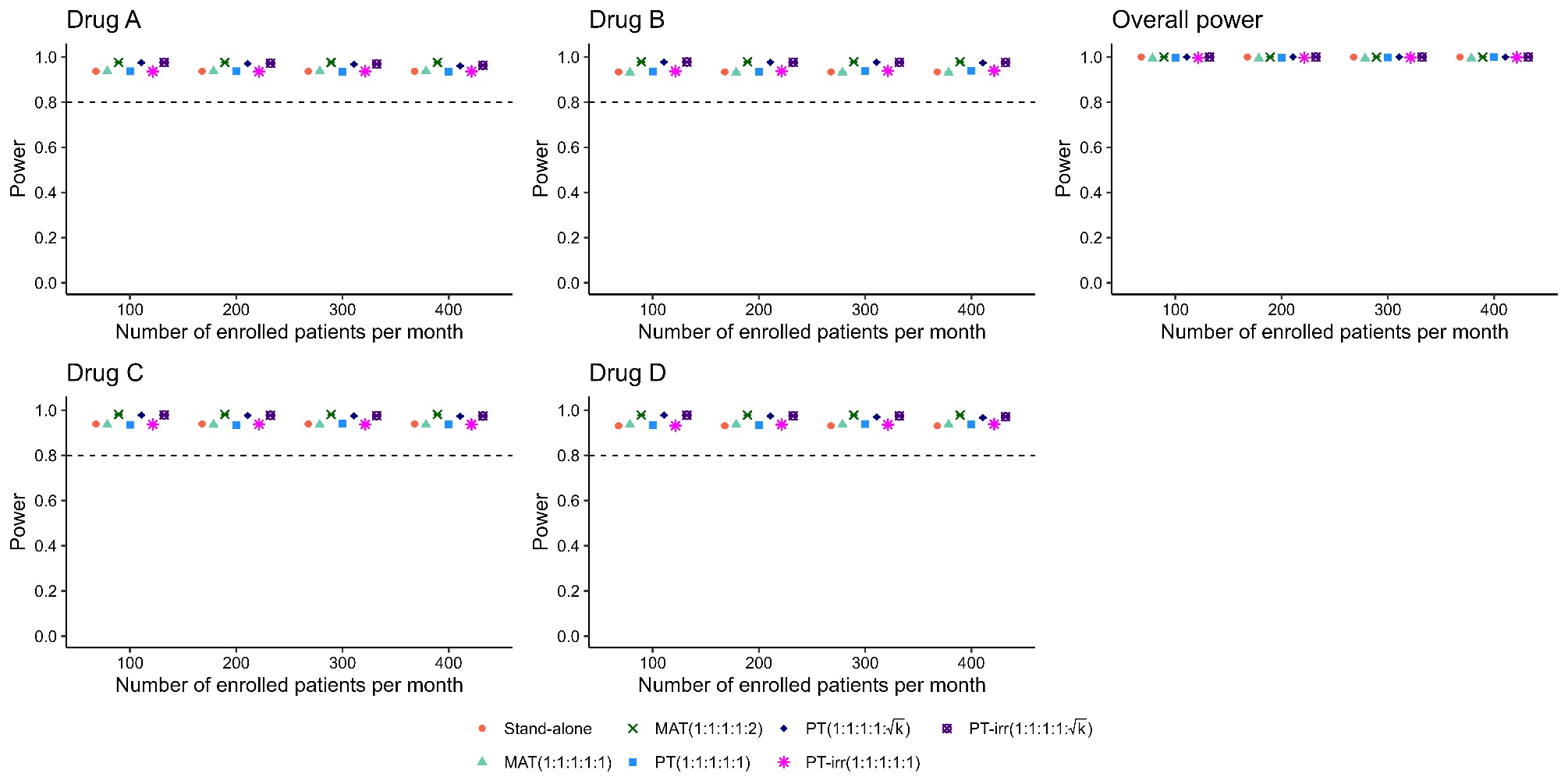


(c1) ($R_{P}, R_{D}$) = (10%, 5%) in scenario 1


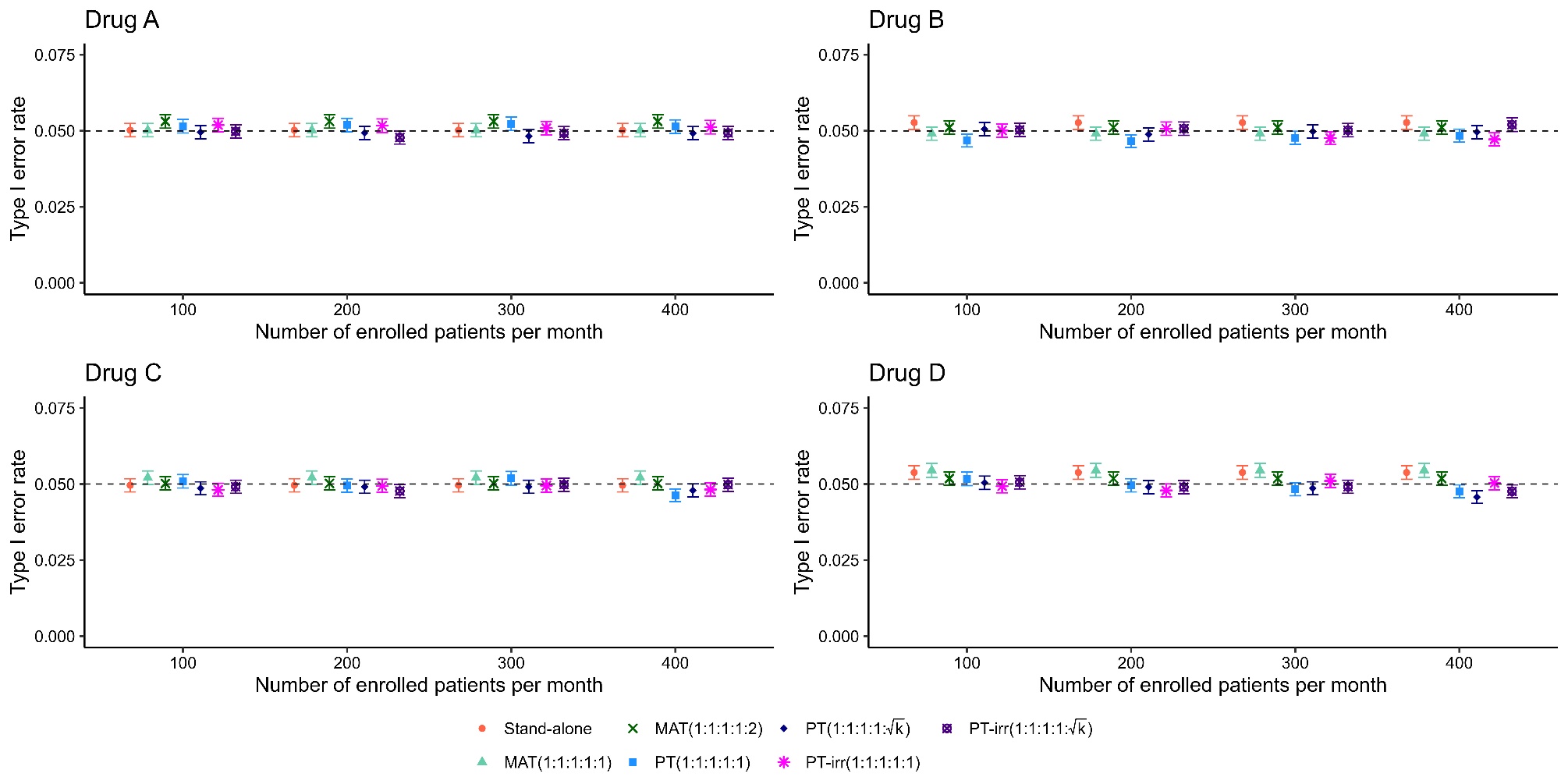


(c2) ($R_{P}, R_{D}$) = (10%, 5%) in scenario 2


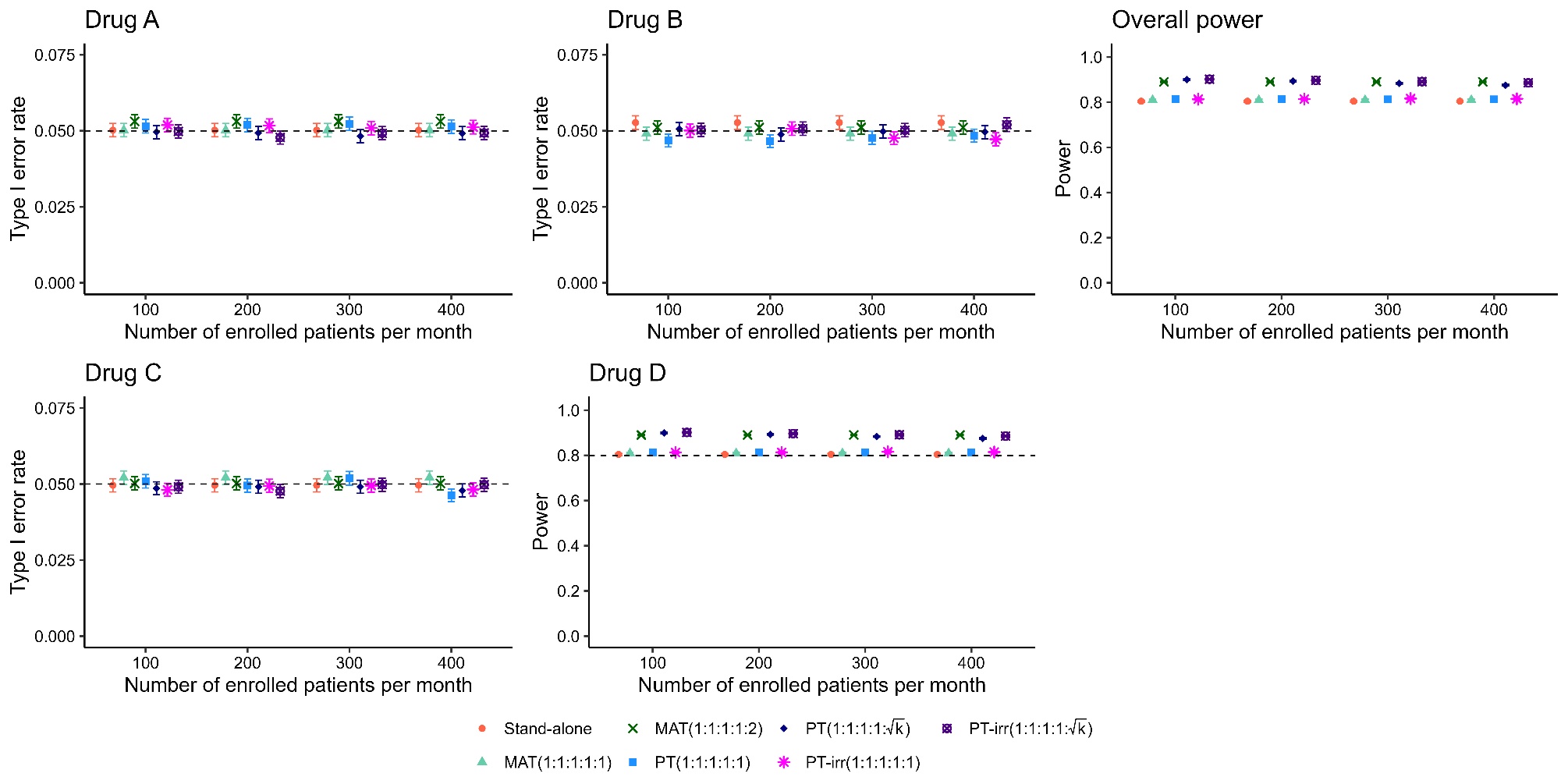


(c3) ($R_{P}, R_{D}$) = (10%, 5%) in scenario 3


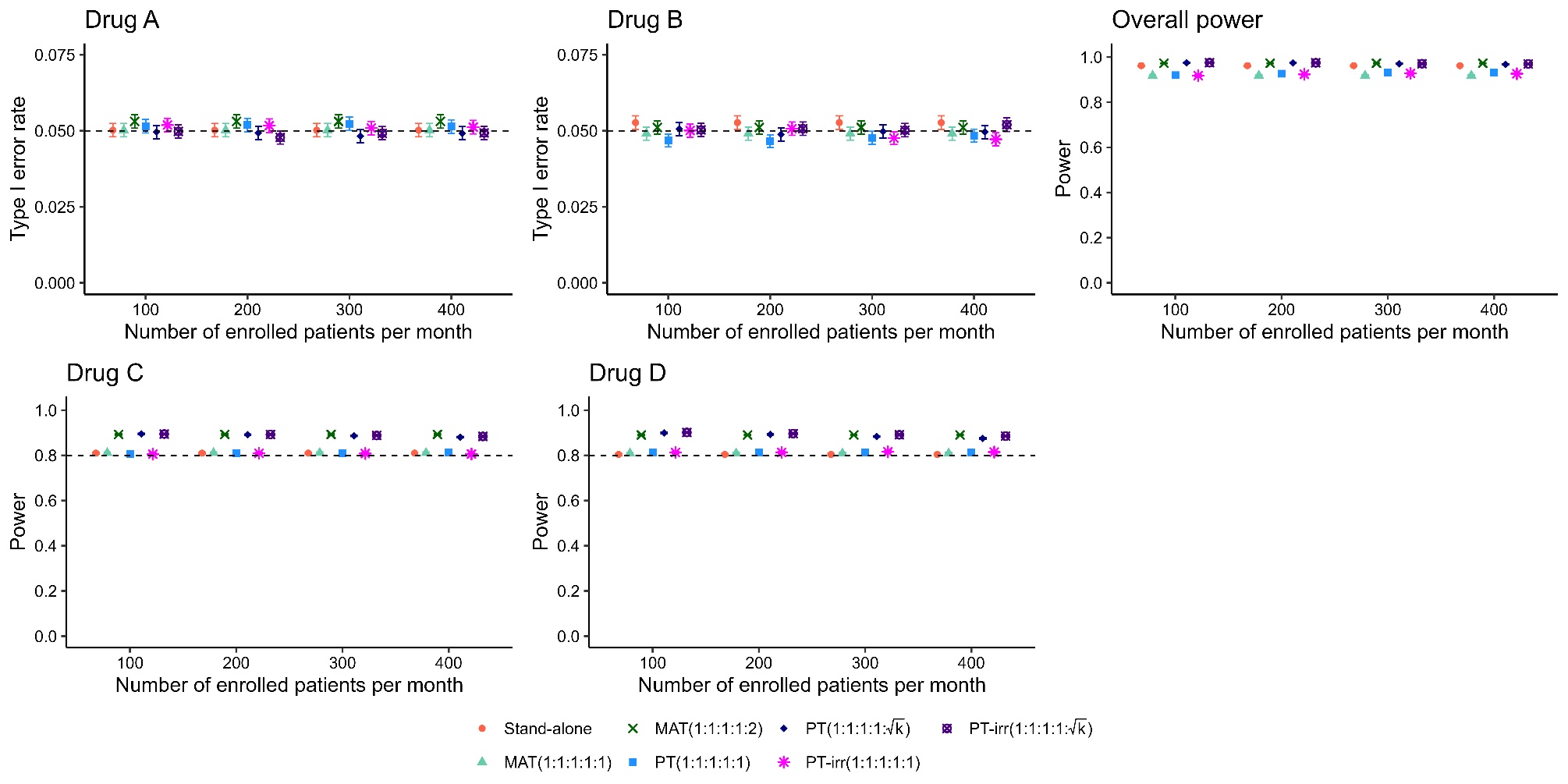


(c4) ($R_{P}, R_{D}$) = (10%, 5%) in scenario 4


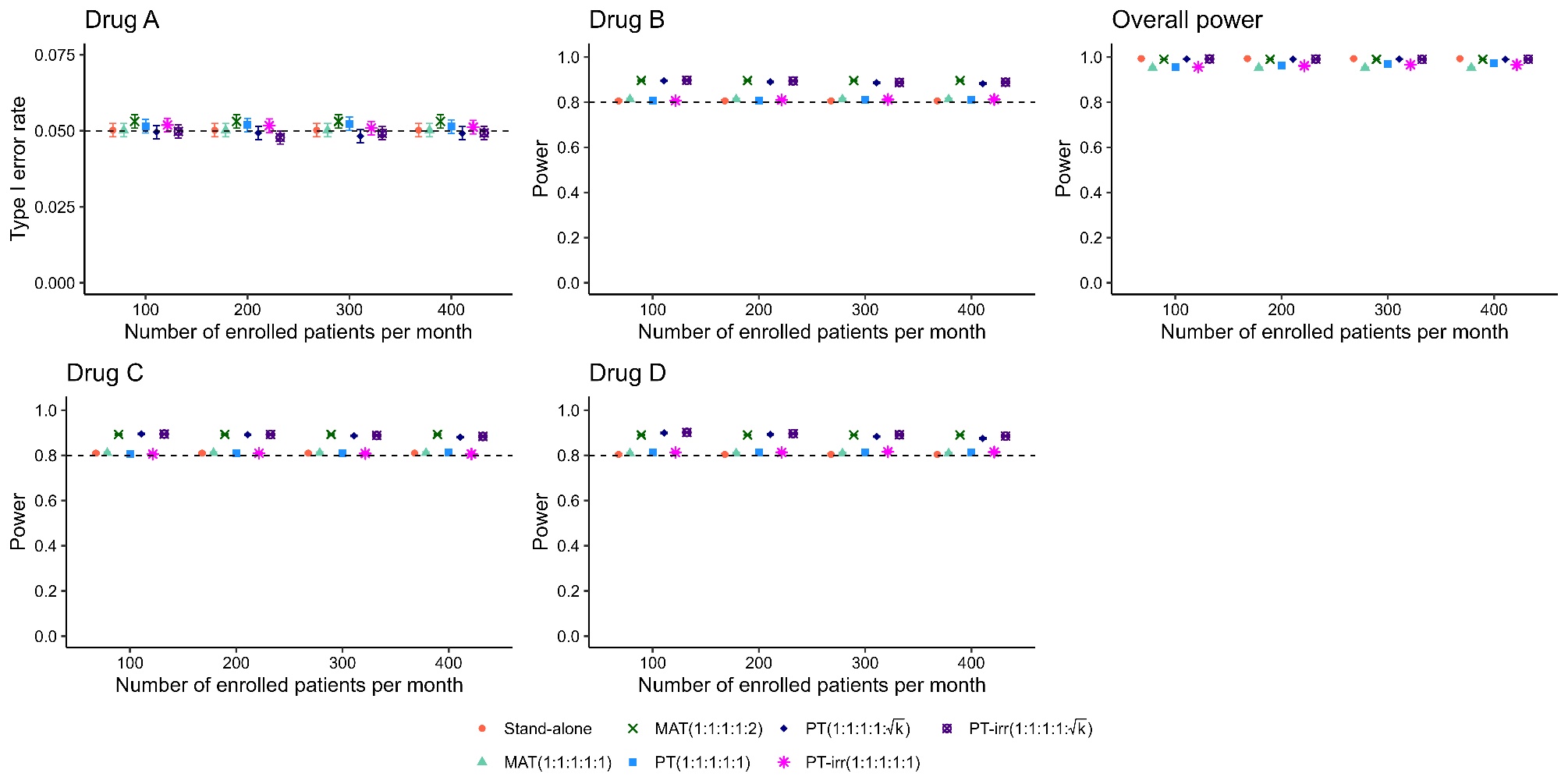


(c5) ($R_{P}, R_{D}$) = (10%, 5%) in scenario 5


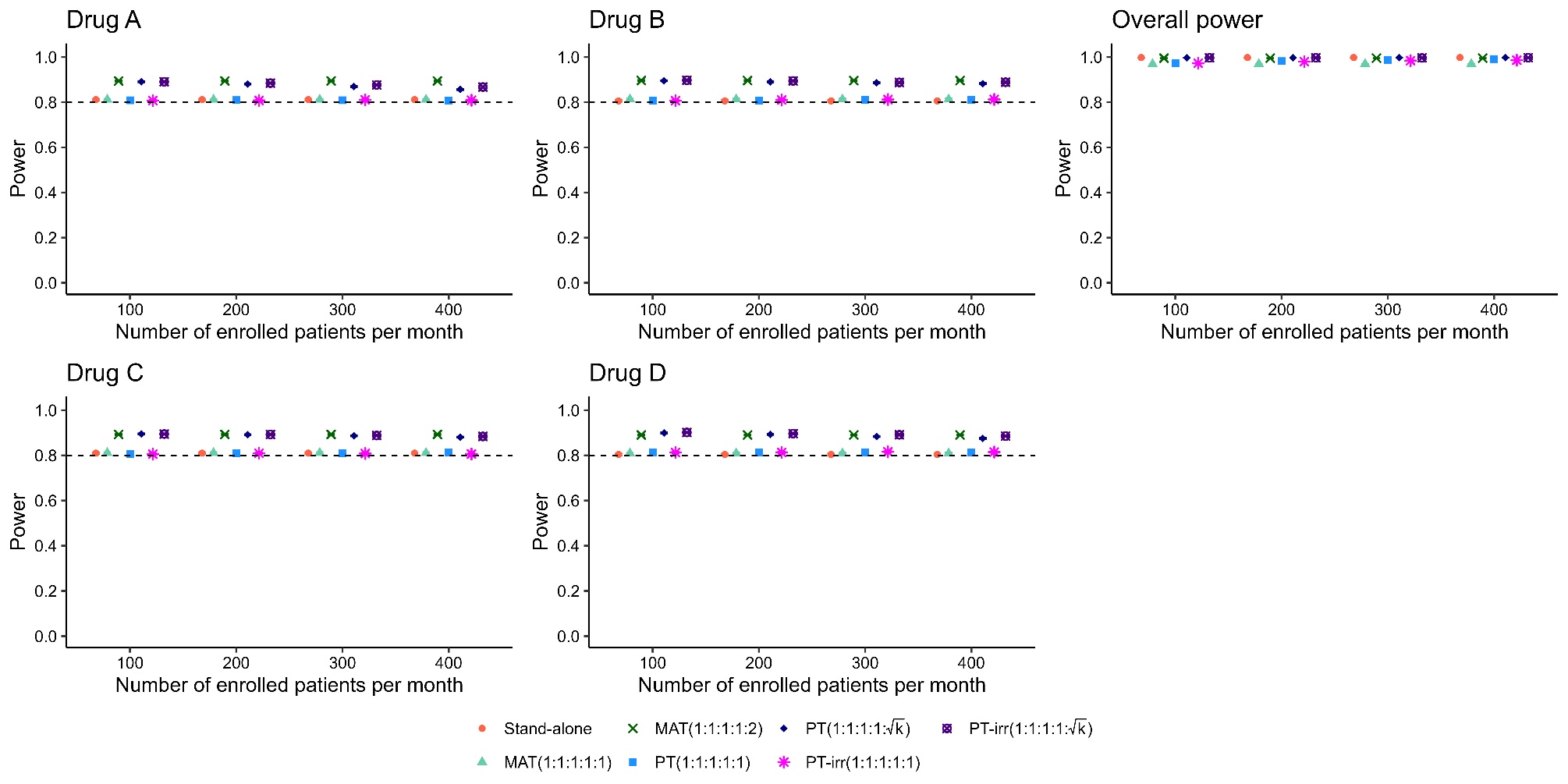


(d1) ($R_{P}, R_{D}$) = (12.5%, 7.5%) in scenario 1


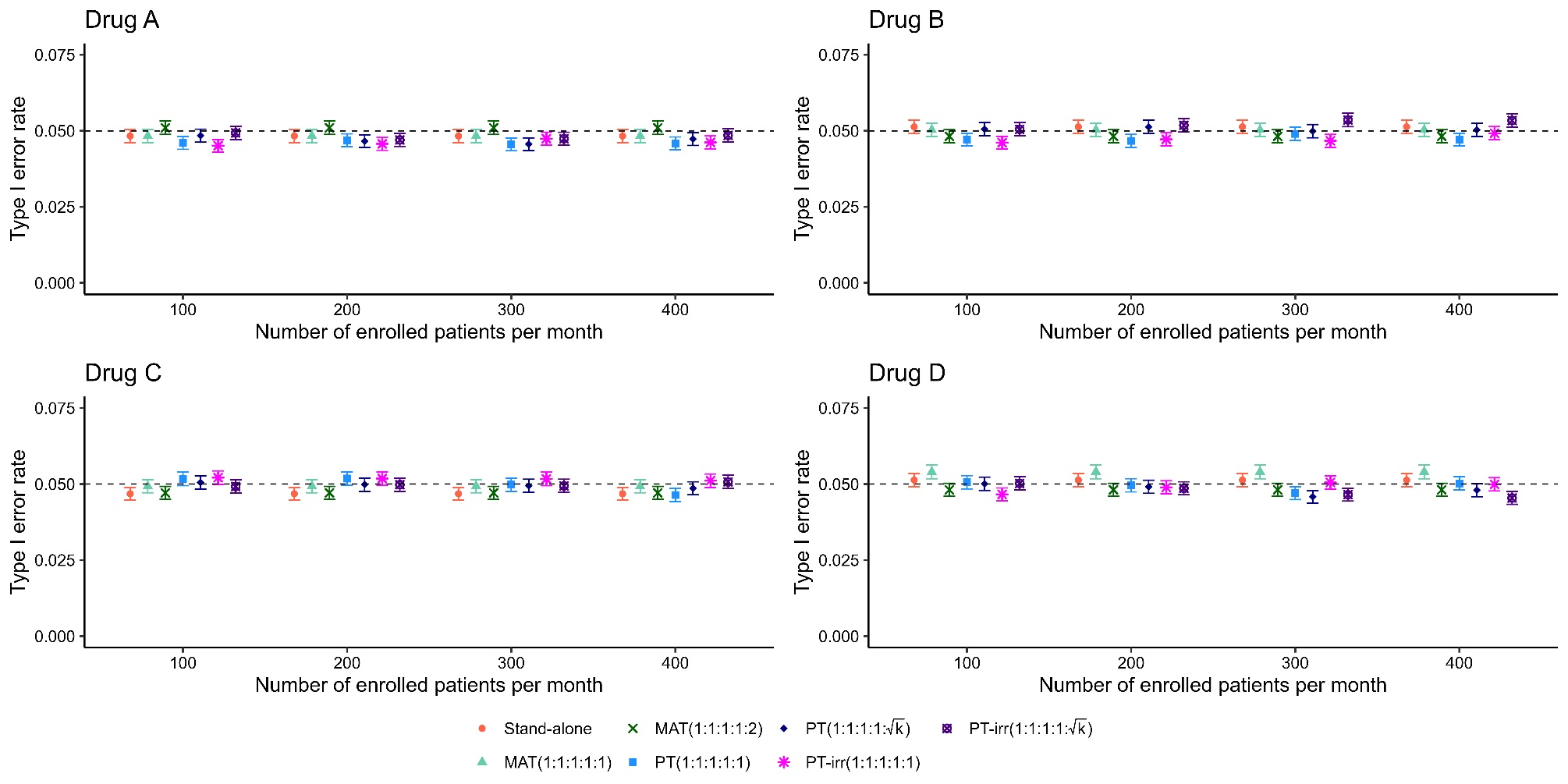


(d2) ($R_{P}, R_{D}$) = (12.5%, 7.5%) in scenario 2


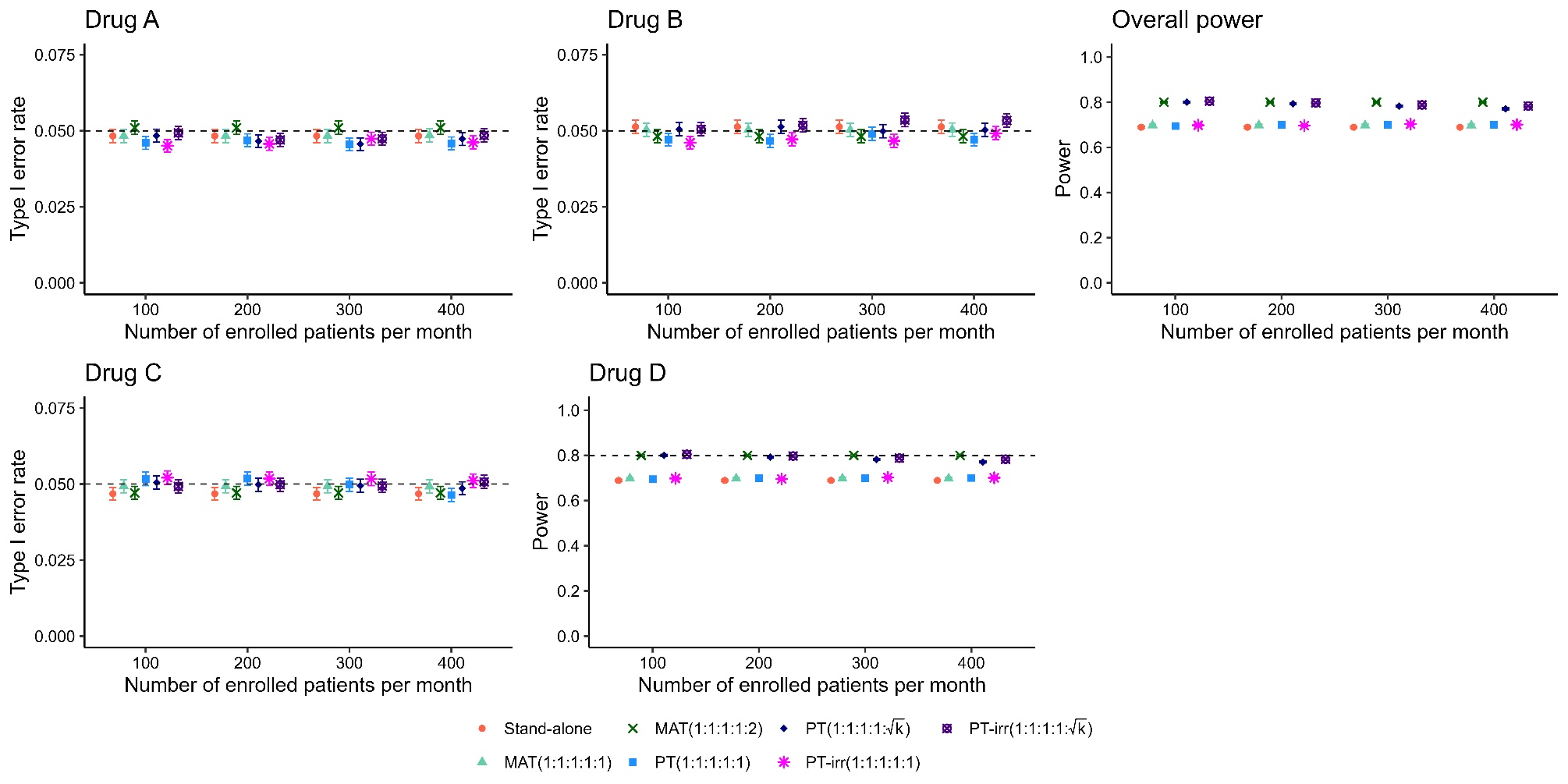


(d3) ($R_{P}, R_{D}$) = (12.5%, 7.5%) in scenario 3


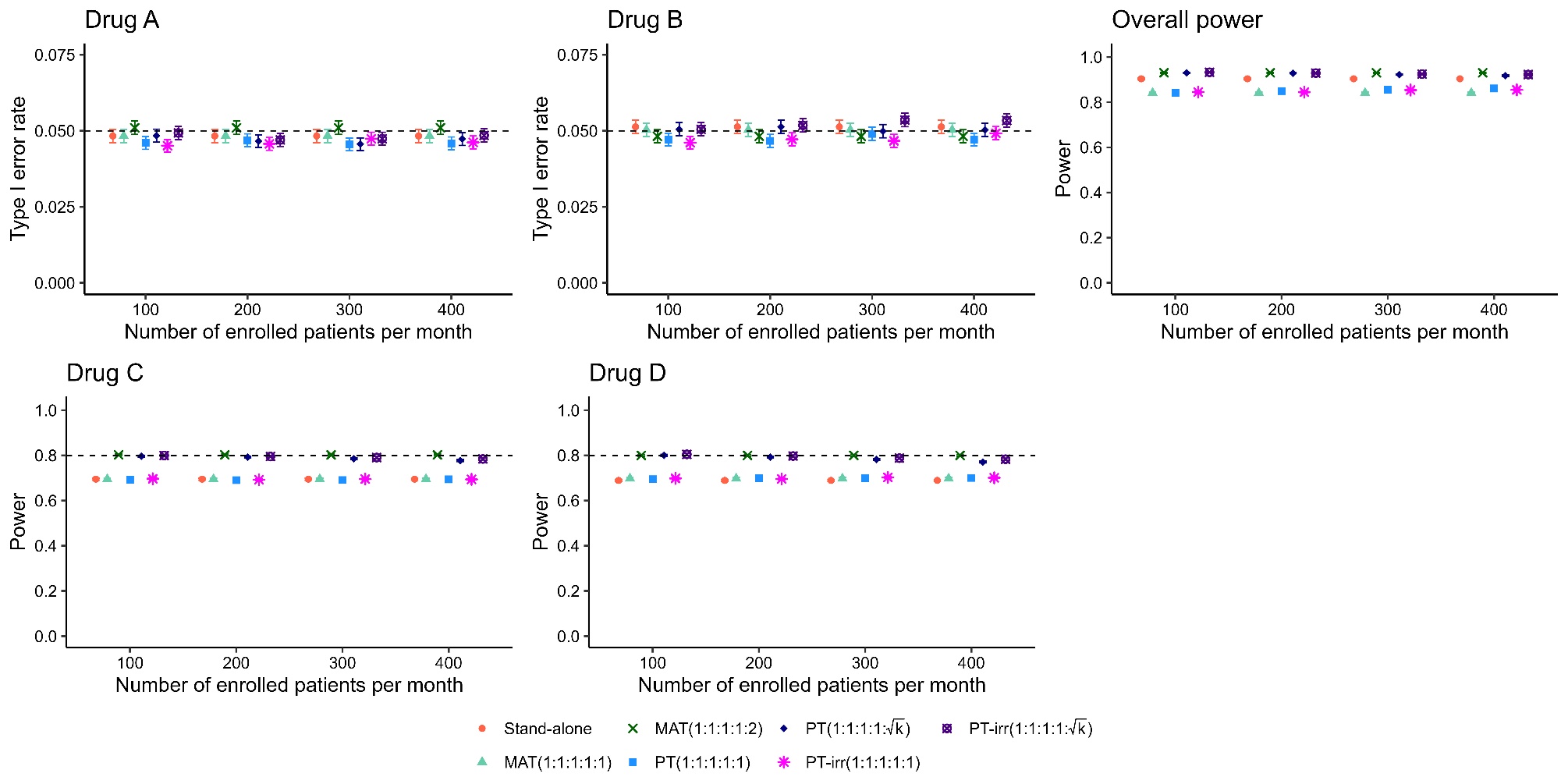


(d4) ($R_{P}, R_{D}$) = (12.5%, 7.5%) in scenario 4


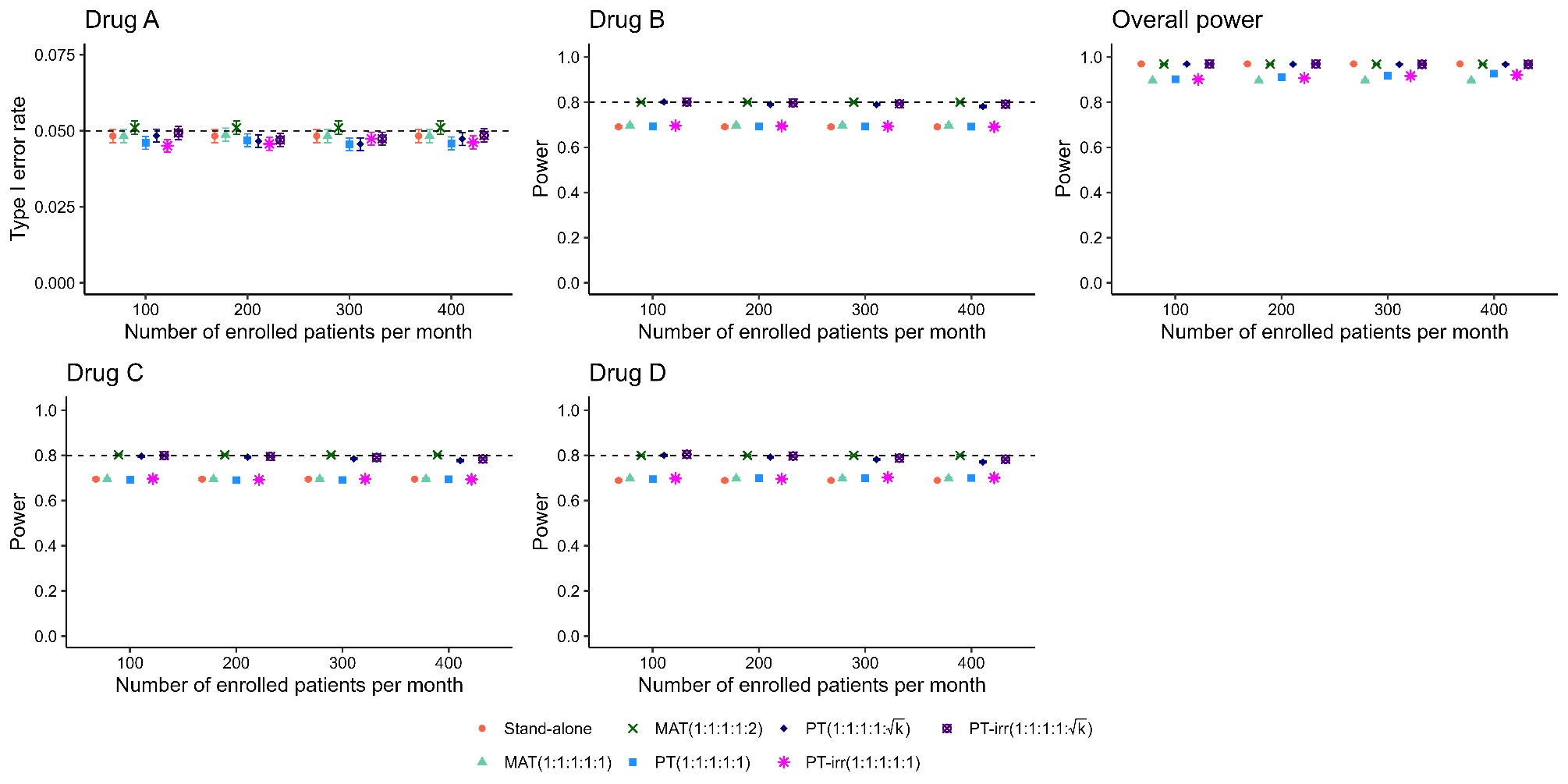


(d5) ($R_{P}, R_{D}$) = (12.5%, 7.5%) in scenario 5


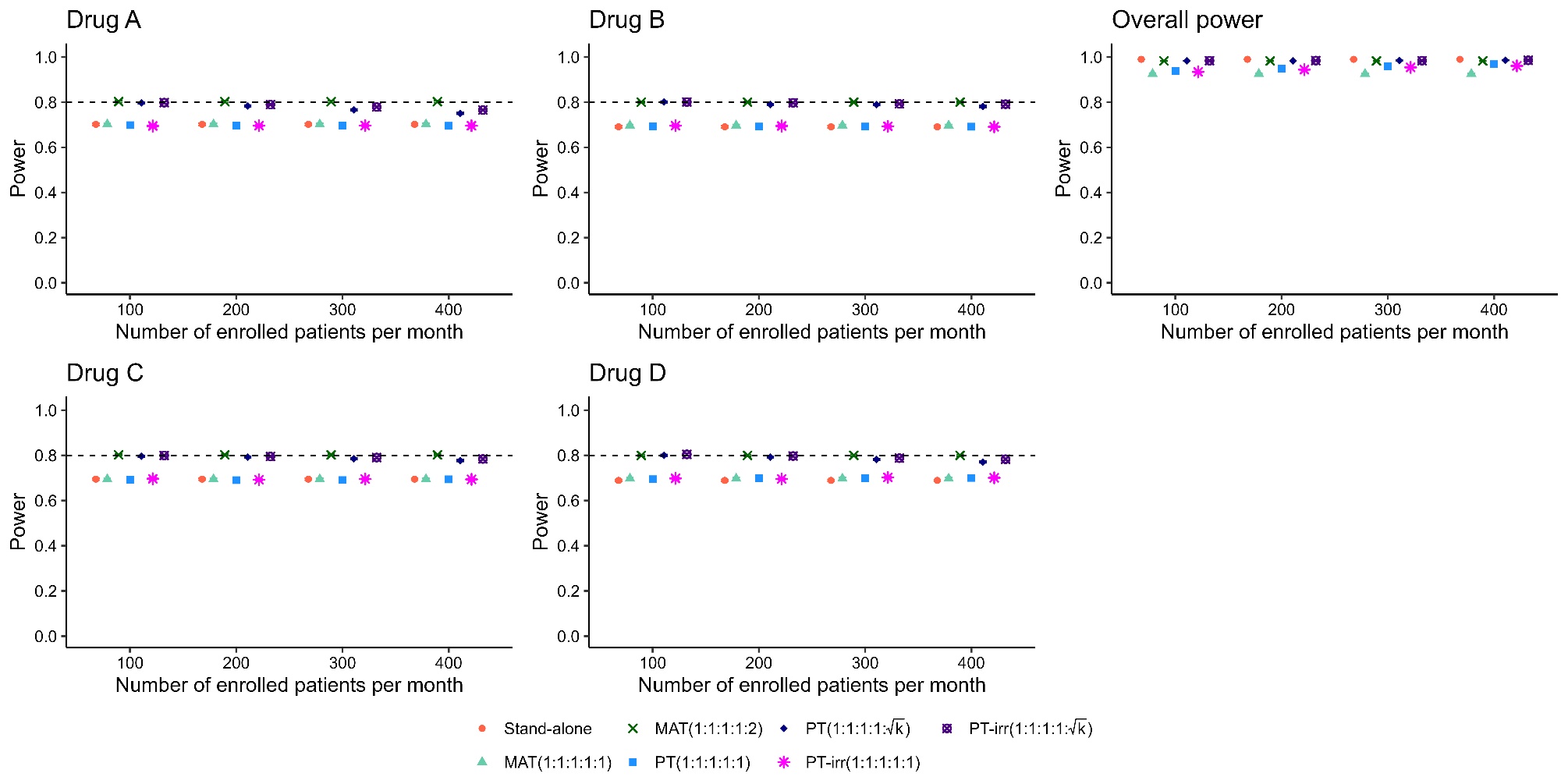


(e1) ($R_{P}, R_{D}$) = (15%, 10%) in scenario 1


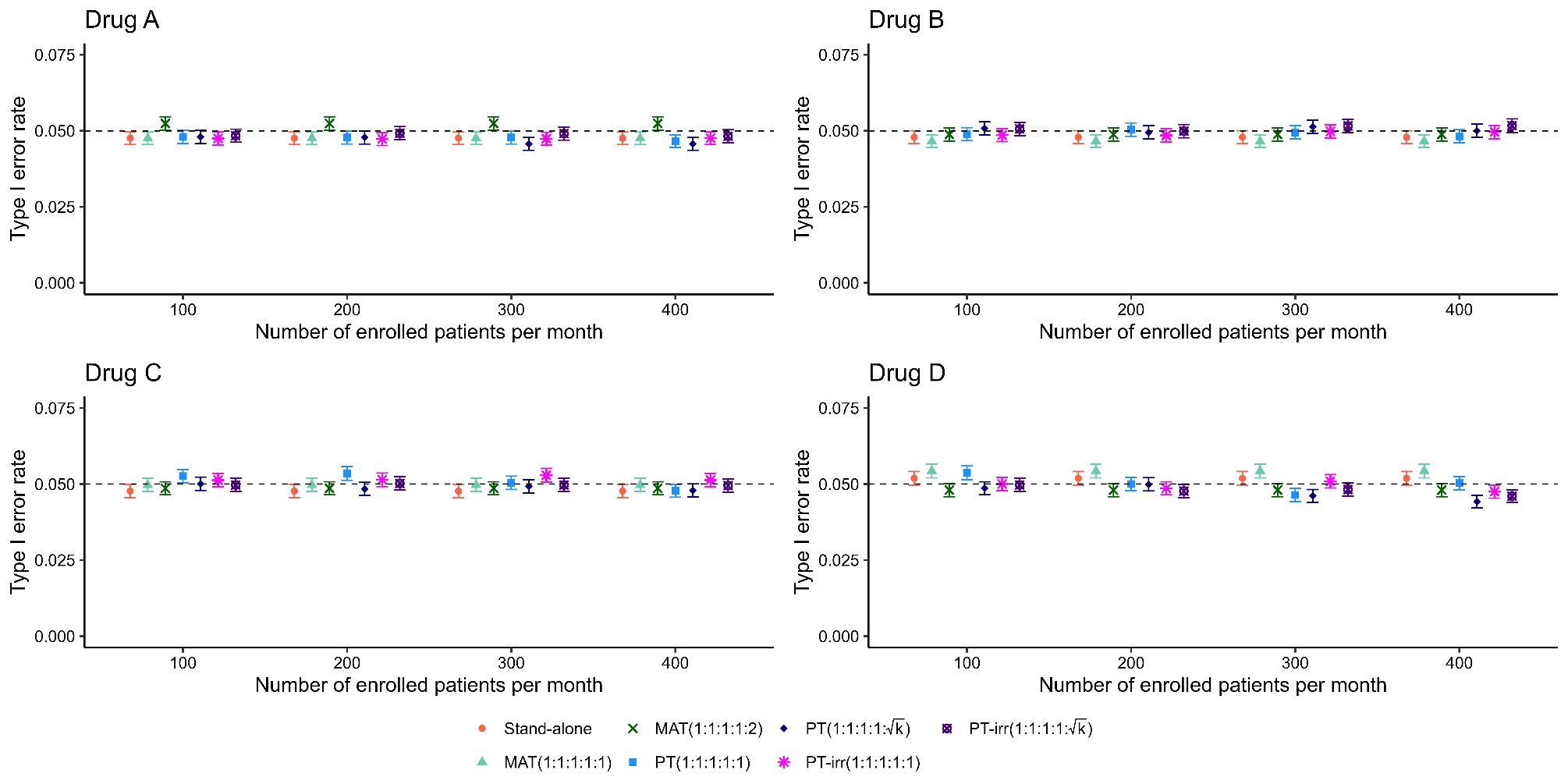


(e2) ($R_{P}, R_{D}$) = (15%, 10%) in scenario 2


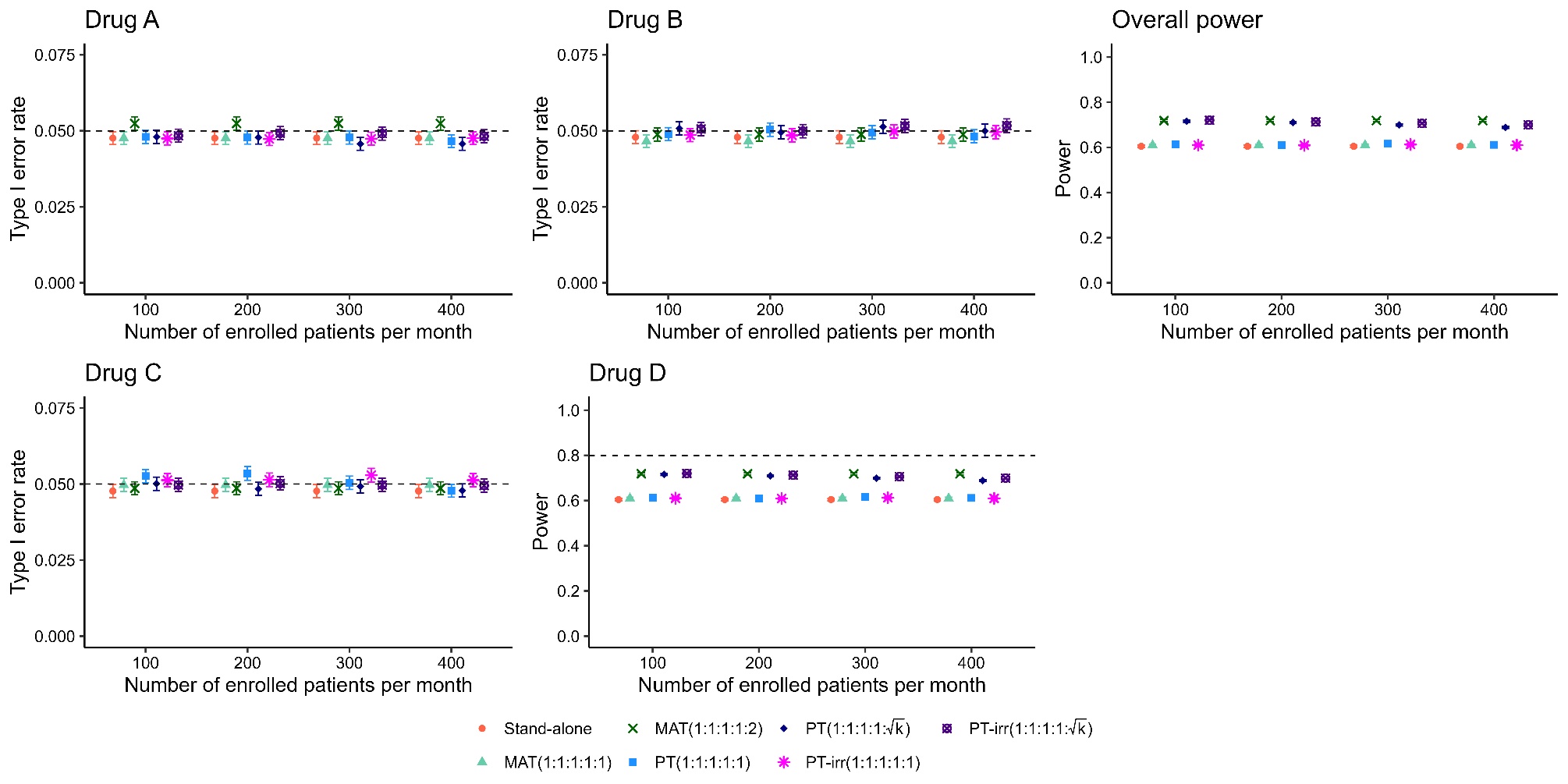


(e3) ($R_{P}, R_{D}$) = (15%, 10%) in scenario 3


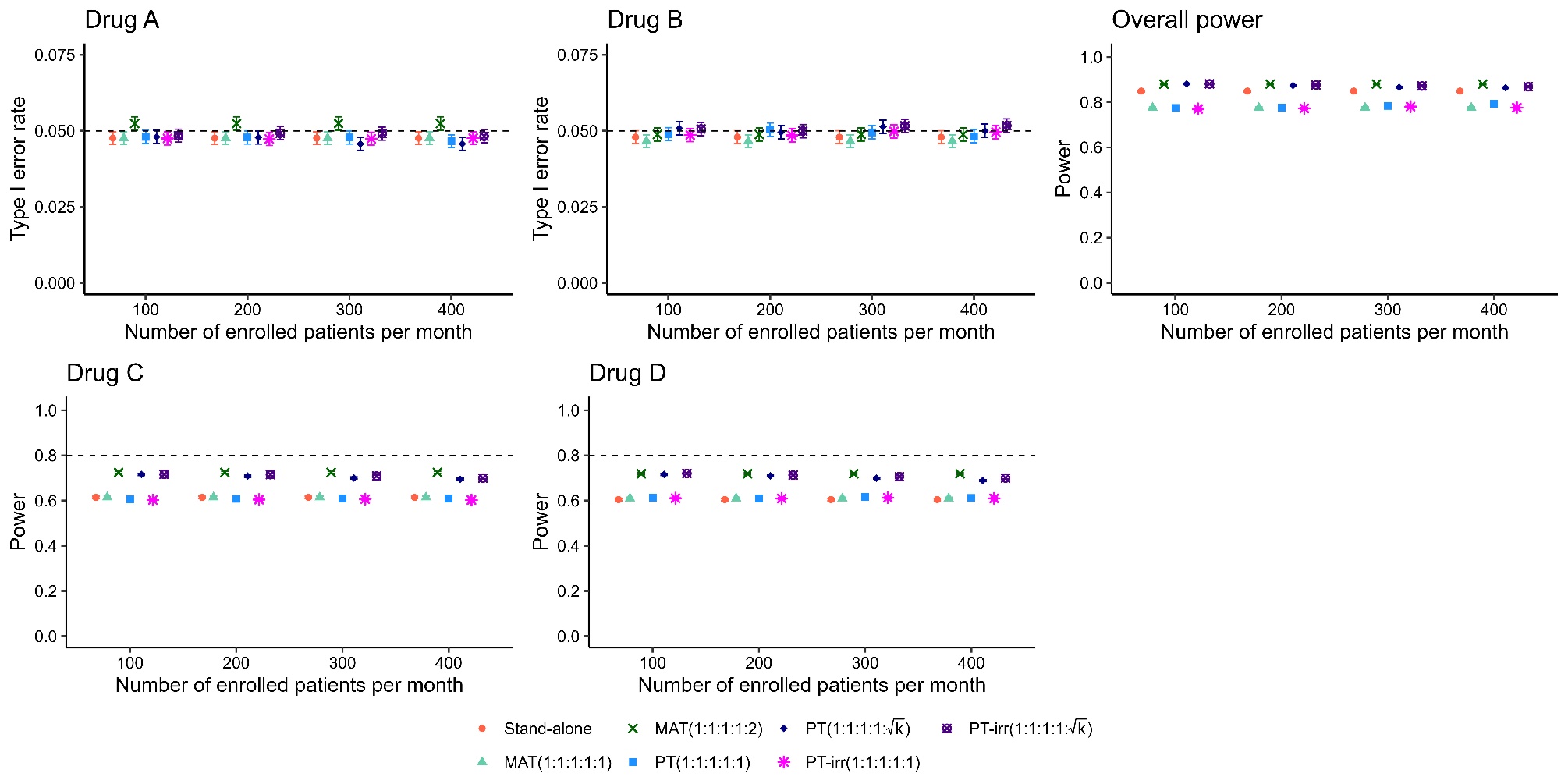


(e4) ($R_{P}, R_{D}$) = (15%, 10%) in scenario 4


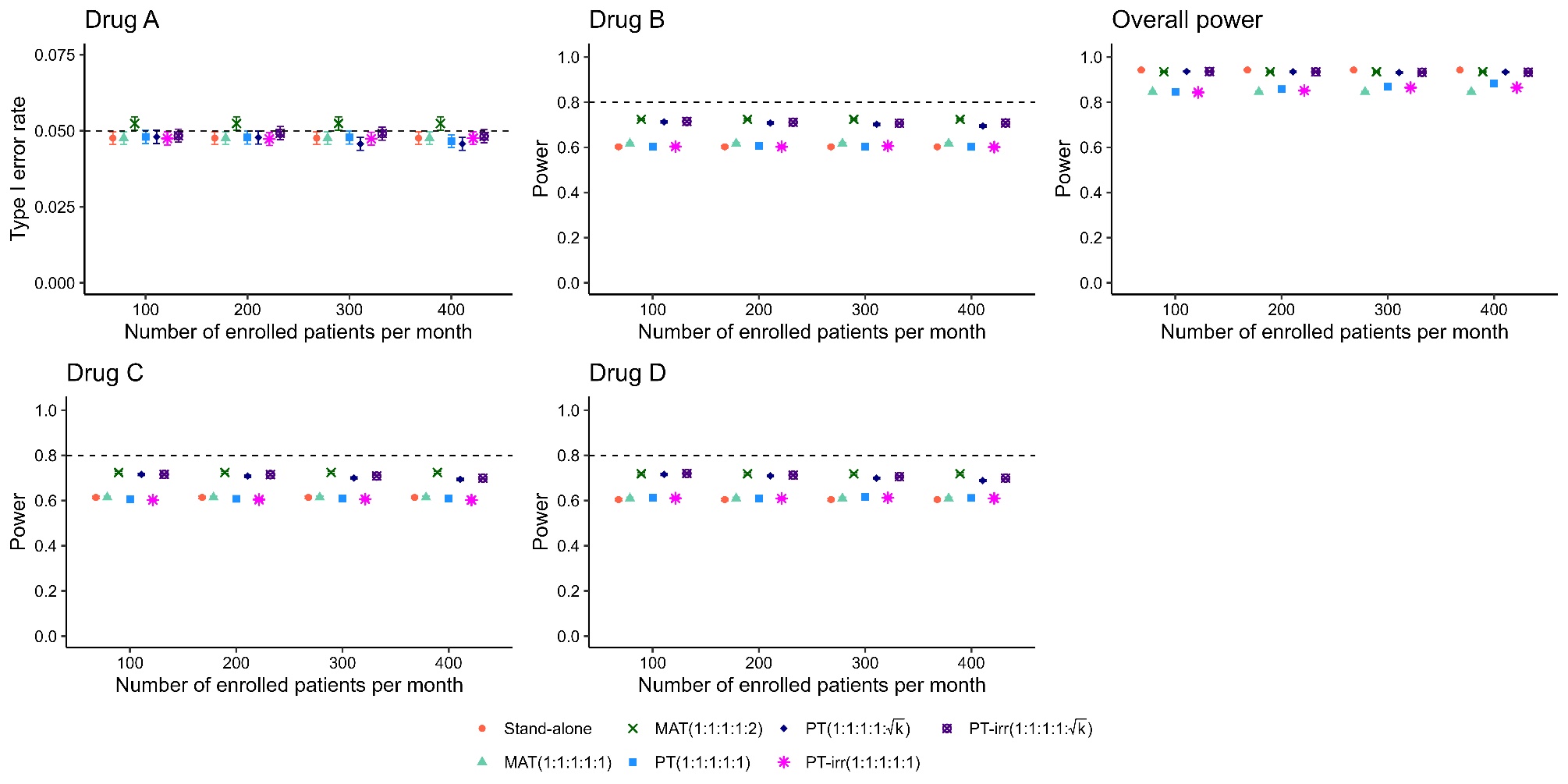


(e5) ($R_{P}, R_{D}$) = (15%, 10%) in scenario 5


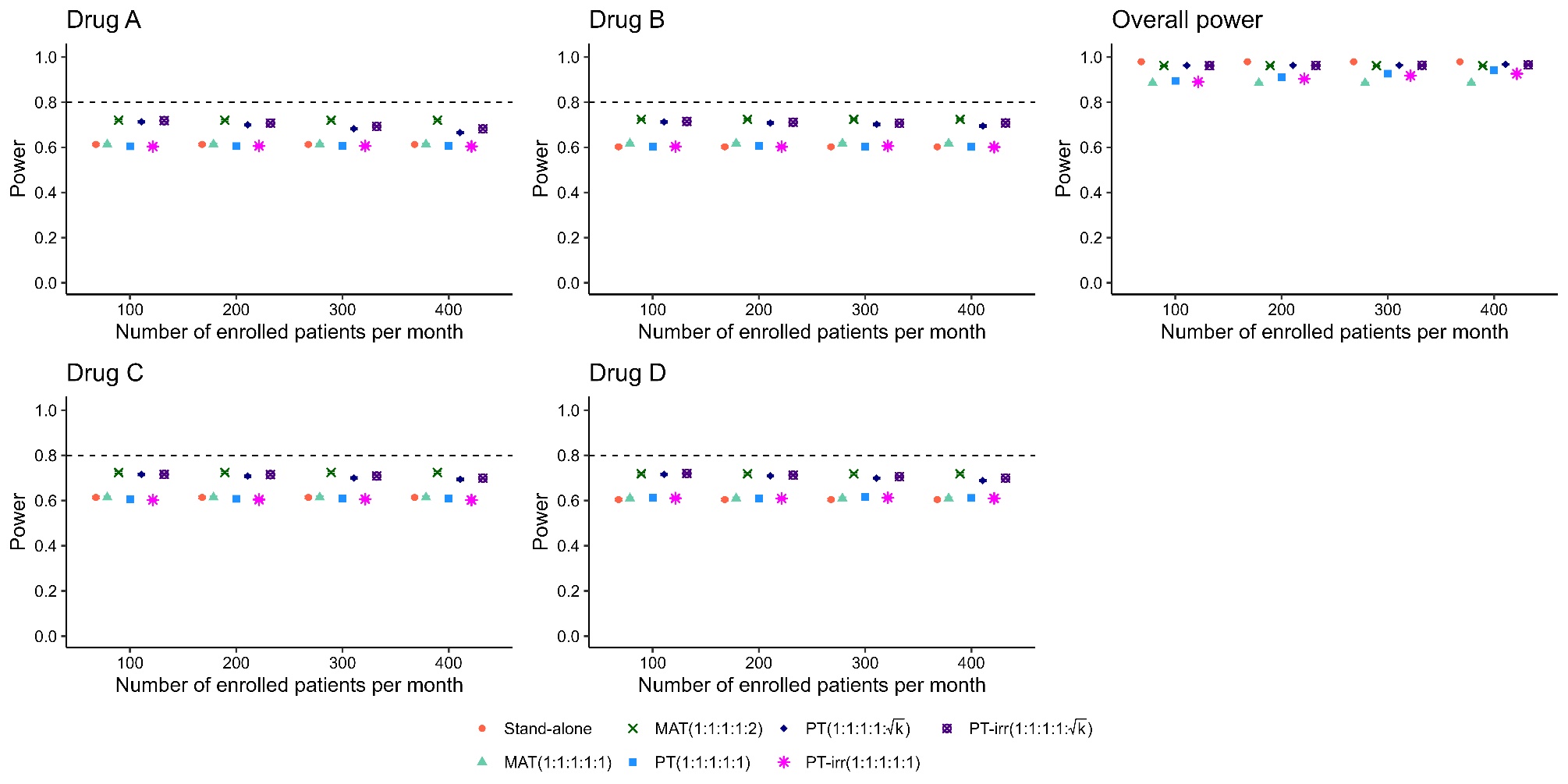


**Figure S2.** Difference in the type I error rate (or power) for four drug groups between PT(1:1:1:1: $\sqrt{k}$) and PT(1:1:1:1:1) (i.e., PT(1:1:1:1: $\sqrt{k}$) − PT(1:1:1:1:1)) for the corresponding increase in total number of patients in the placebo group of PT(1:1:1:1:$\sqrt{k}$), assuming that the true mortality rate for the placebo group was 5%, 7.5%, 10%, 12.5%, and 15%. The Monte Carlo standard errors for the difference in the type I error rate (or power) are presented as error bars.
Abbreviation: PT, platform trials with drugs added every month; TIE, type I error.


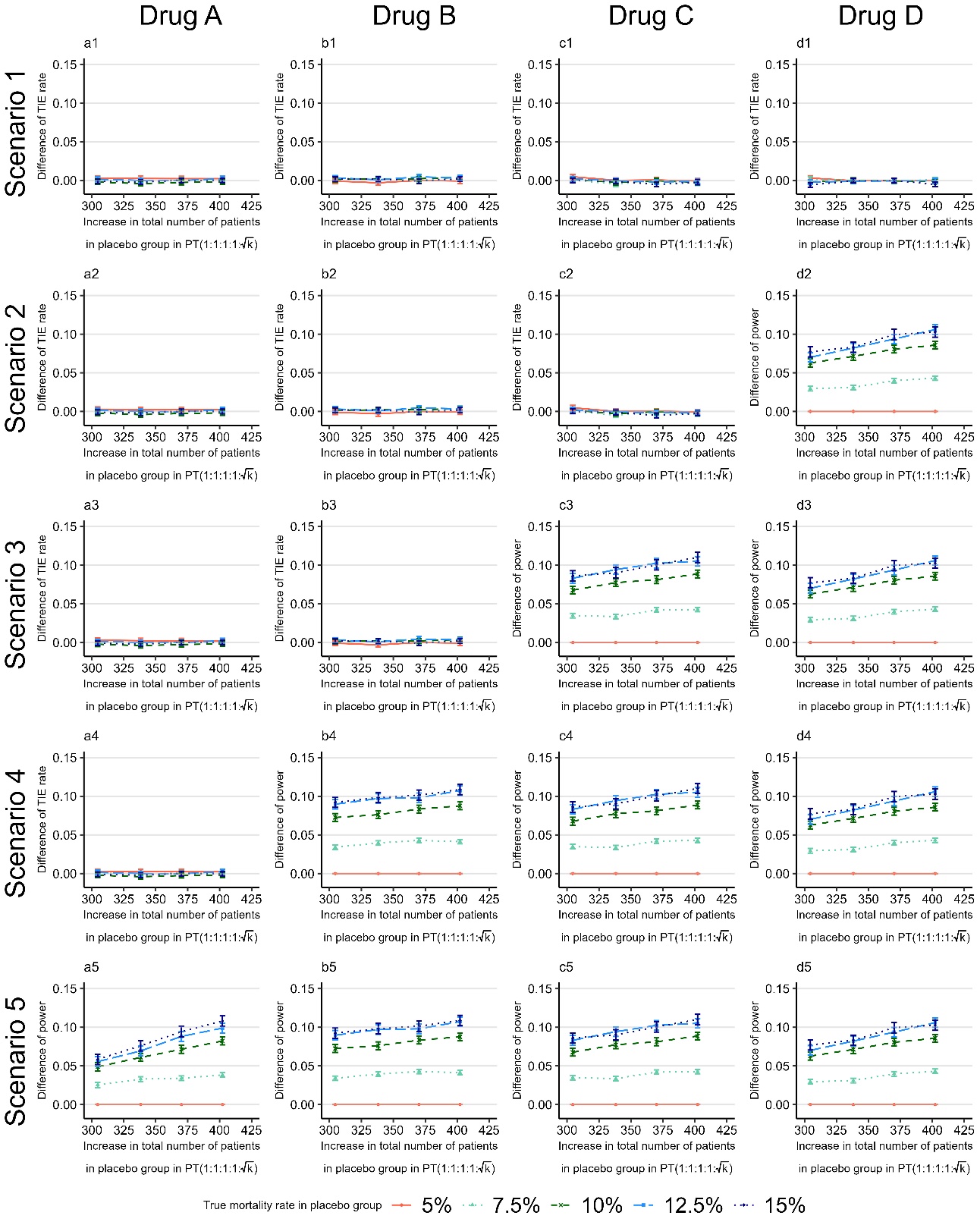


**Figure S3.** Difference in the average type I error rate (or power) across four drug groups between PT-irr(1:1:1:1:$\sqrt{k}$) and PT-irr(1:1:1:1:1) (i.e., PT-irr(1:1:1:1:$\sqrt{k}$) − PT-irr(1:1:1:1:1)) for the corresponding increase in total number of patients in the placebo group of PT-irr(1:1:1:1:$\sqrt{k}$), assuming that the true mortality rate for the placebo group was 5%, 7.5%, 10%, 12.5%, and 15%. The Monte Carlo standard errors for the difference in the average type I error rate (or power) are presented as error bars.
Abbreviations: PT-irr, platform trials with irregular intervals for drug addition; TIE, type I error.


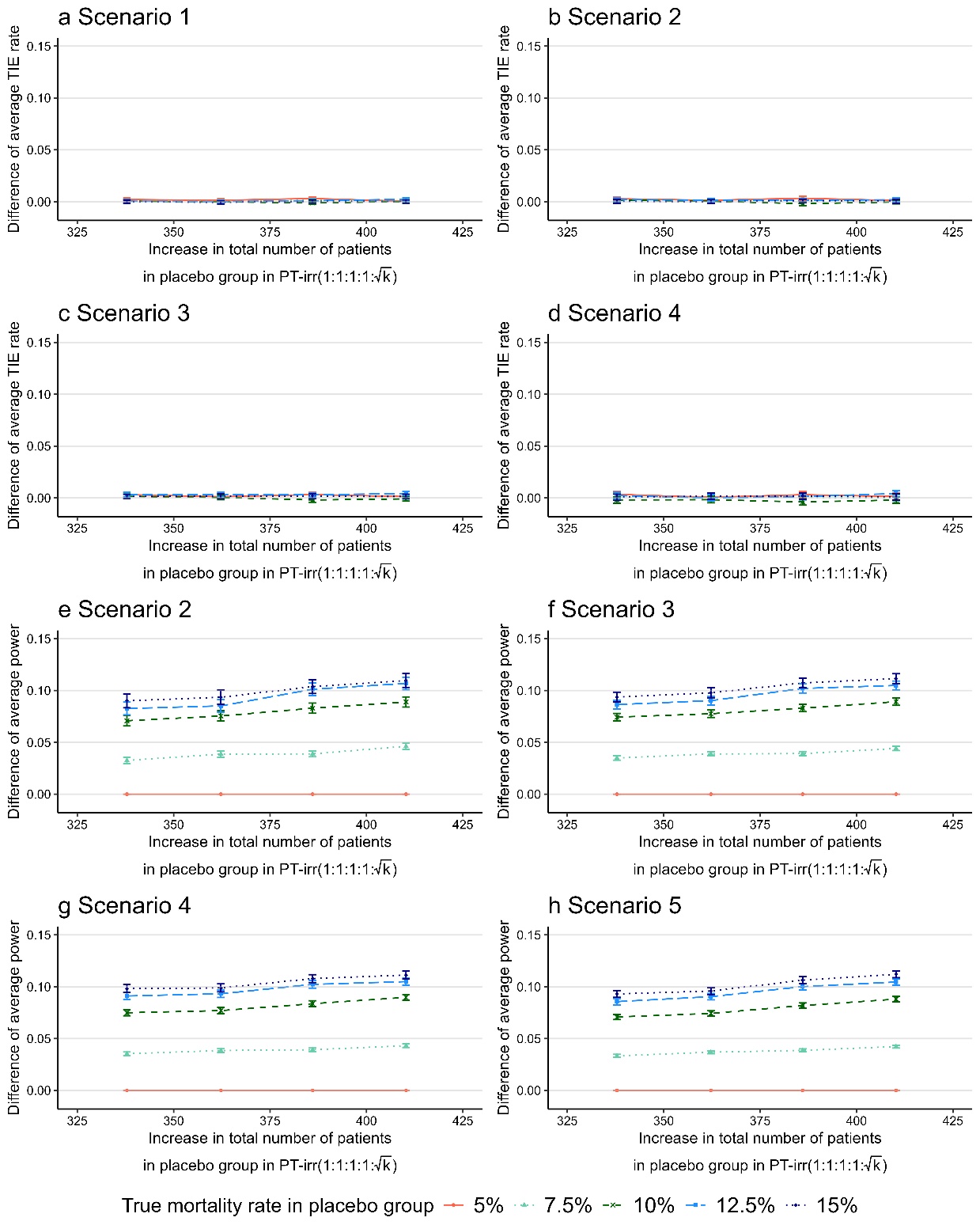


**Figure S4.** Difference in the average type I error rate and average group-specific power across the drug groups included in the PT between PT(1:1:1:1:$\sqrt{k}$) and PT(1:1:1:1:1) (e.g., PT(1:1:1:1:$\sqrt{k}$) − PT(1:1:1:1:1)) and between PT-irr(1:1:1:1:$\sqrt{k}$) and PT-irr(1:1:1:1:1) (e.g., PT-irr(1:1:1:1:$\sqrt{k}$) − PT-irr(1:1:1:1:1)) when the total number of drugs was three, four, or five with an average enrollment of 200 patients per month and the true mortality rate for the placebo group, $R_{P}$, ranging from 5% to 15%. The Monte Carlo standard errors for the difference in the average type I error rate (or group-specific power) are presented as error bars. The number of scenarios depends on the number of drugs included in the PT (see Tables 2, S1, and S2). Therefore, the results for PTs with three or four drugs are not displayed in scenarios 5 and 6.

Abbreviations: PT, platform trials with drugs added every month; PT-irr, platform trials with irregular intervals for drug addition; TIE, type I error.


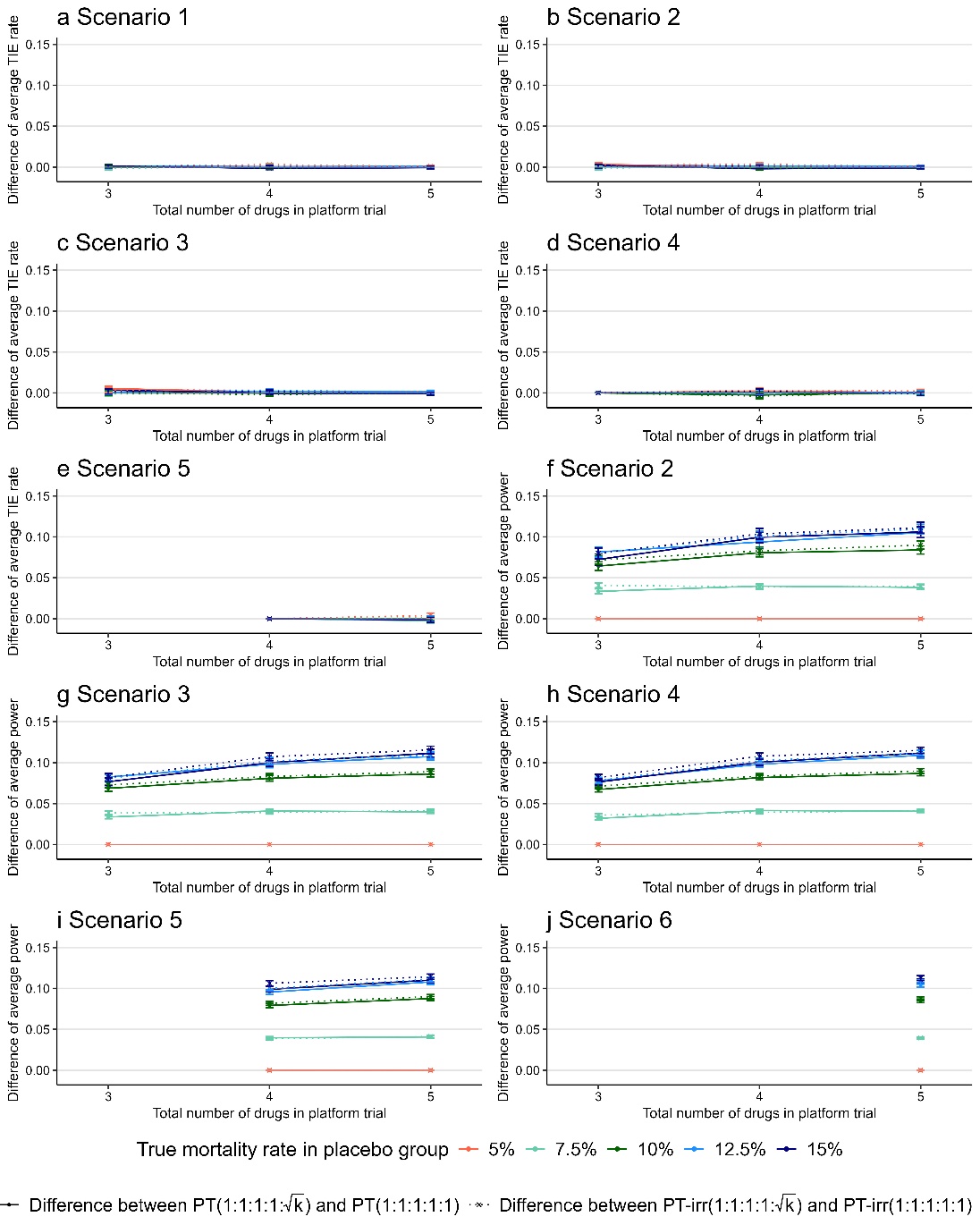


**Figure S5.** Difference in the average type I error rate and average group-specific power across the drug groups included in the PT between PT(1:1:1:1:$\sqrt{k}$) and PT(1:1:1:1:1) (e.g., PT(1:1:1:1:$\sqrt{k}$) − PT(1:1:1:1:1)) and between PT-irr(1:1:1:1:$\sqrt{k}$) and PT-irr(1:1:1:1:1) (e.g., PT-irr(1:1:1:1:$\sqrt{k}$) − PT-irr(1:1:1:1:1)) when the total number of drugs was three, four, or five with an average enrollment of 300 patients per month and the true mortality rate for the placebo group, $R_{P}$, ranging from 5% to 15%. The Monte Carlo standard errors for the difference in the average type I error rate (or group-specific power) are presented as error bars. The number of scenarios depends on the number of drugs included in the PT (see Tables 2, S1, and S2). Therefore, the results for PTs with three or four drugs are not displayed in scenarios 5 and 6.

Abbreviations: PT, platform trials with drugs added every month; PT-irr, platform trials with irregular intervals for drug addition; TIE, type I error.


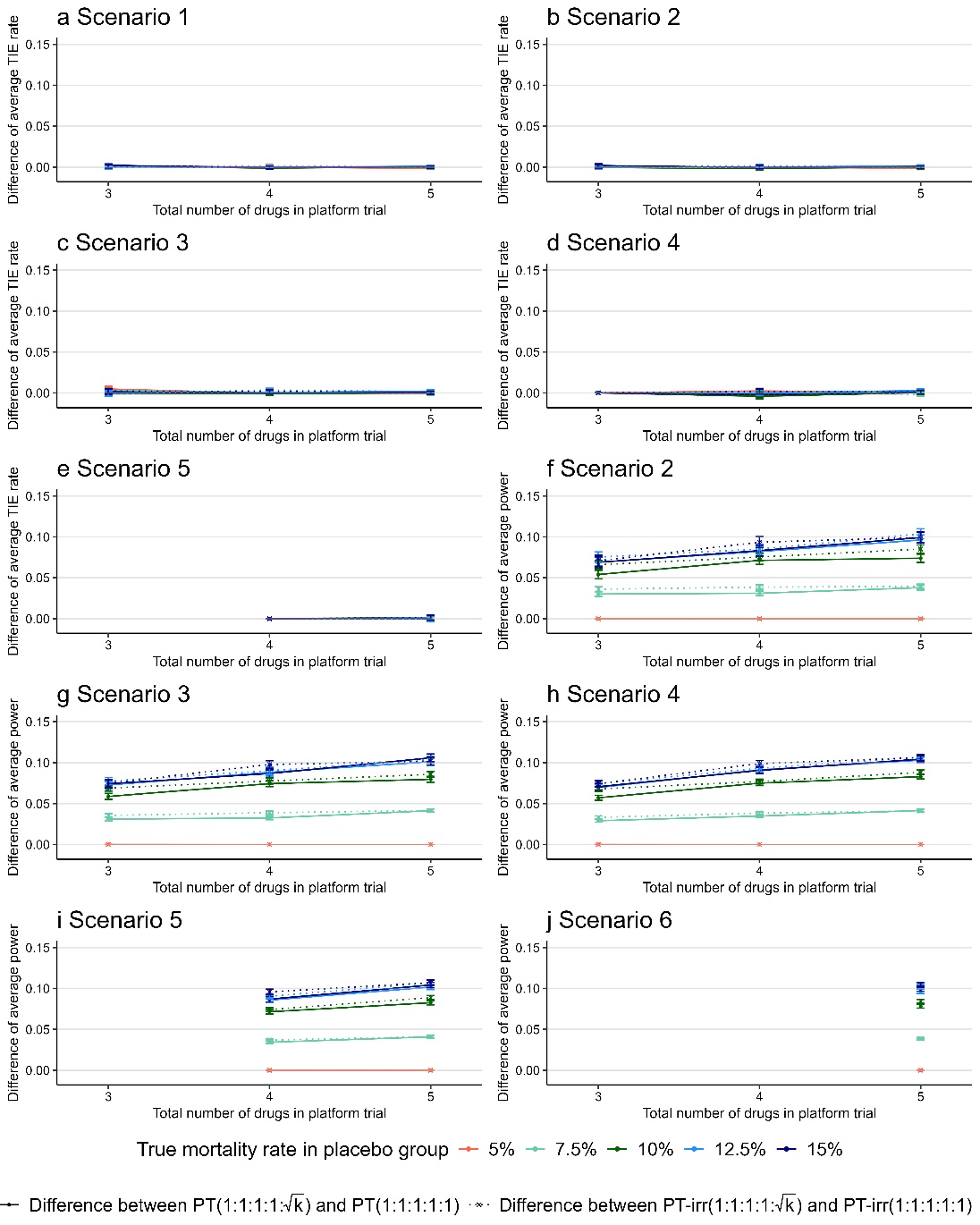


**Figure S6.** Difference in the average type I error rate and average group-specific power across the drug groups included in the PT between PT(1:1:1:1:$\sqrt{k}$) and PT(1:1:1:1:1) (e.g., PT(1:1:1:1:$\sqrt{k}$) − PT(1:1:1:1:1)) and between PT-irr(1:1:1:1:$\sqrt{k}$) and PT-irr(1:1:1:1:1) (e.g., PT-irr(1:1:1:1:$\sqrt{k}$) − PT-irr(1:1:1:1:1)) when the total number of drugs was three, four, or five with an average enrollment of 400 patients per month and the true mortality rate for the placebo group, $R_{P}$, ranging from 5% to 15%. The Monte Carlo standard errors for the difference in the average type I error rate (or group-specific power) are presented as error bars. The number of scenarios depends on the number of drugs included in the PT (see Tables 2, S1, and S2). Therefore, the results for PTs with three or four drugs are not displayed in scenarios 5 and 6.

Abbreviations: PT, platform trials with drugs added every month; PT-irr, platform trials with irregular intervals for drug addition; TIE, type I error.


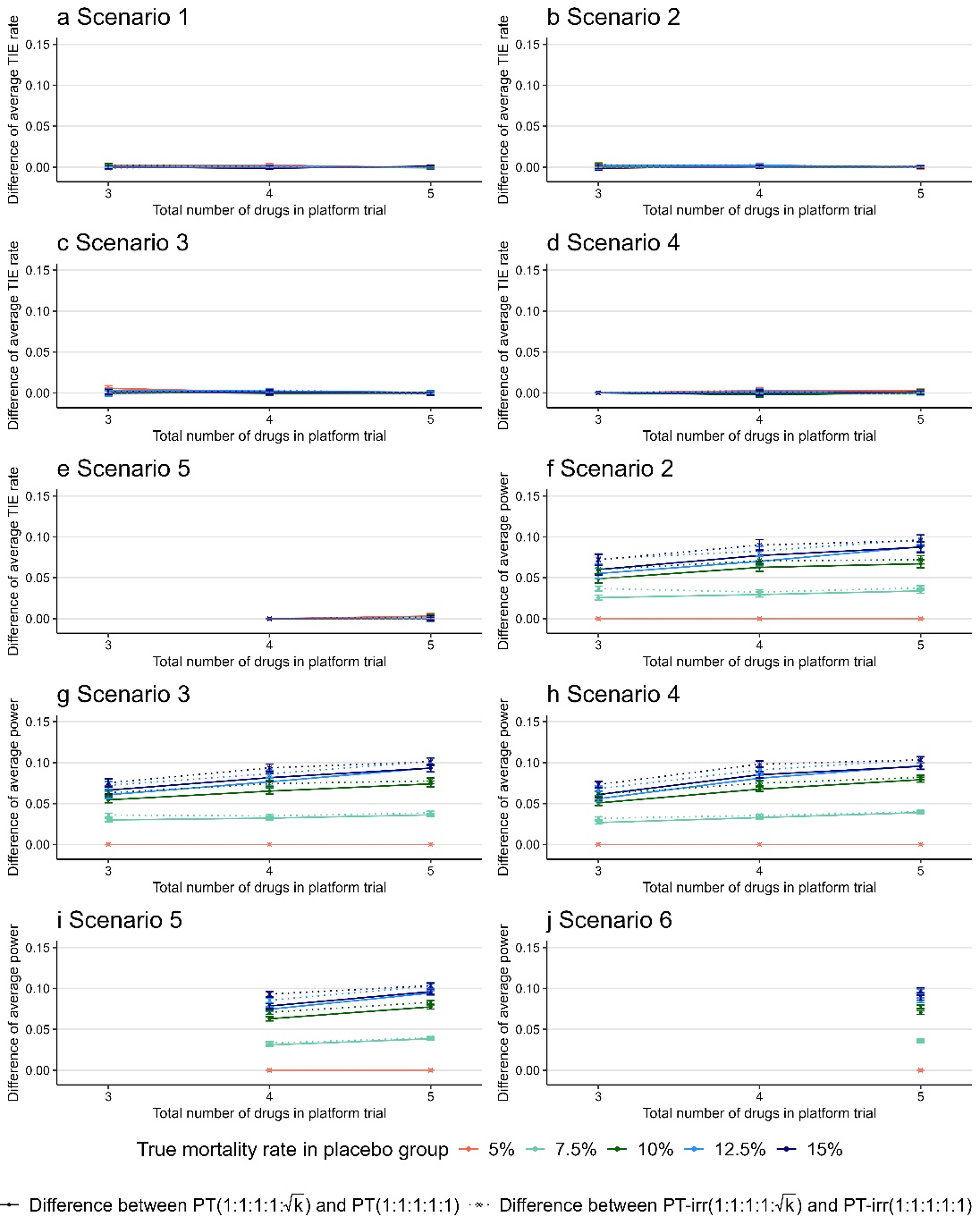

Supplement: Multimedia component 1 [file mmc1.docx]
